# CPVL promotes glioma progression via STAT1 pathway inhibition through interactions with the BTK/p300 axis

**DOI:** 10.1172/jci.insight.146362

**Published:** 2021-12-22

**Authors:** Hui Yang, Xiaocen Liu, Xiaolong Zhu, Xueqin Li, Lan Jiang, Min Zhong, Mengying Zhang, Tianbing Chen, Mingzhe Ma, Xiuming Liang, Kun Lv

**Affiliations:** 1 Key Laboratory of Non-coding RNA Transformation Research of Anhui Higher Education Institution,; 2 Central Laboratory, The First Affiliated Hospital of Wannan Medical College, Yijishan Hospital of Wannan Medical College,; 3 Non-coding RNA Research Center of Wannan Medical College, Yijishan Hospital, and; 4 Department of Nuclear Medicine, The First Affiliated Hospital of Wannan Medical College, Yijishan Hospital of Wannan Medical College, Wuhu, Anhui Province, PR China.; 5 Department of Gastric Surgery, Fudan University Shanghai Cancer Center, Shanghai, PR China.; 6 Biomolecular Medicine, Clinical Research Center, Department of Laboratory Medicine, Karolinska Institutet, Stockholm, Sweden.

**Keywords:** Oncology, Oncogenes

## Abstract

CPVL (carboxypeptidase, vitellogenic-like) is a serine carboxypeptidase that was first characterized in human macrophages. However, the function of CPVL remains unclear in a variety of tumors. The quantitative PCR (qPCR), Western blotting, and IHC assays were utilized to measure the CPVL expression. CPVL was significantly upregulated in glioma cells and tissues compared with normal cells and tissues, respectively. Moreover, high CPVL expression was correlated with advanced clinical grade and poor prognosis. Silencing of CPVL promoted glioma cell apoptosis, and it inhibited cell proliferation and tumorigenicity in vitro and in vivo. Ingenuity Pathway Analysis (IPA) demonstrated that CPVL silencing activated the IFN-γ/STAT1 signaling pathway, thereby inducing glioma cell apoptosis. Mechanistically, immunopurification, mass spectrometry, IP, and glutathione S-transferase (GST) pull-down experiments elucidated that CPVL physically interacts with Bruton’s tyrosine kinase (BTK) and downregulates the STAT1 phosphorylation through promoting p300-mediated STAT1 acetylation. Our findings reveal the crucial role of CPVL in promoting the progression of glioma through suppressing STAT1 phosphorylation. CPVL might serve as a potential prognostic biomarker and therapeutic target for the treatment of glioma.

## Graphical abstract

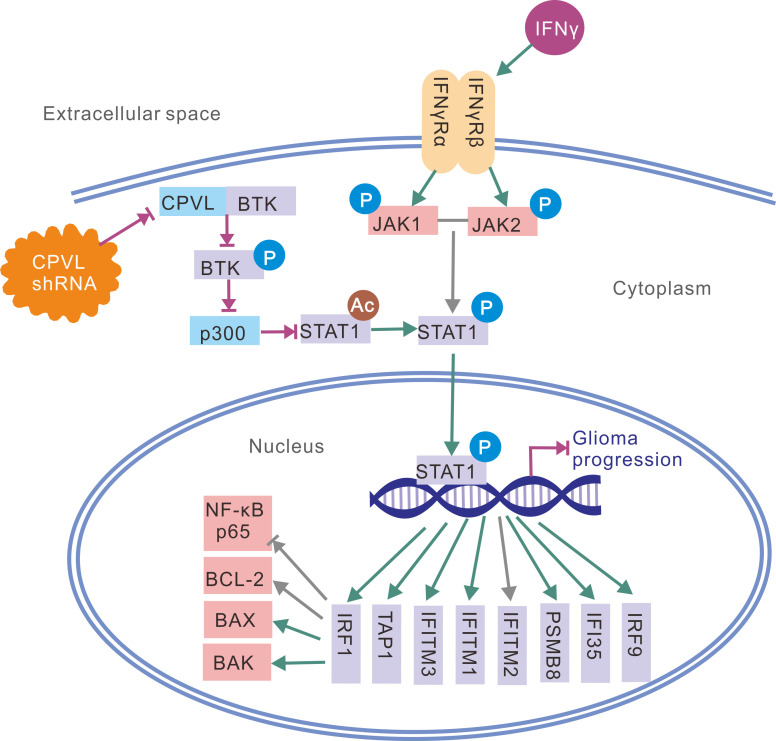

## Introduction

Glioma is one of the most common types of primary brain tumors in adults and represents one of the most aggressive and lethal human cancer types (1–3). The World Health Organization (WHO) classifies glioma into 4 grades: I–IV (4). Despite the advances in early detection, most of the patients are at grade IV glioblastomas (GBM) at the time of diagnosis; thus, the prognosis of these patients remains poor (5, 6). The median survival period of GBM is only 12–15 months following diagnosis (7). There are unmet needs for improved therapeutic options for GBM (8). Although dramatic efforts have been put into identifying the molecules that are critical for glioma cell invasion and proliferation, very few were characterized up to date (9–11).

Increasing evidence indicates that inhibition of glioma cell apoptosis is an early event that leads to increased tumorigenicity (12, 13). Thus, it is of great clinical value to identify potential early-induced apoptosis biomarkers to improve the diagnosis and prognostic assessment of glioma (14). Gene chip and microarray expression profiling are technologies that have been extensively employed in the past decade to rapidly compare expression levels of a large number of genes in different samples, making them suitable for gene screening (15). In this study, CPVL (carboxypeptidase, vitellogenic-like) was screened out by microarray expression profiling. CPVL is a serine carboxypeptidase that was first characterized in human macrophages (16). CPVL was first cloned and characterized during a process to search for human macrophage–restricted genes using differential display PCR (16). CPVL expression is notable in various tissues, including spleen, placenta, heart, and kidneys (17, 18). However, the function of CPVL in various tumors, including glioma, has remained unclear until now.

Signal transducer and activator of transcription 1 (STAT1), the first described member of the STAT transcription factor family, is a major transcription factor in the IFN-α/β and IFN-γ signal transduction pathways (19). There are compelling pieces of evidence that STAT1 acts as the tumor suppressor in both the tumor environment and the tumor cells themselves (20, 21). Clinical and experimental studies also showed the abnormal expression and dysfunction of STAT1 in glioma. STAT1 presented a lower expression level in GBM tissue compared with that in normal human brain tissue (22, 23). Overexpression of STAT1 significantly inhibited the glioma cell growth and increased apoptotic cell death (22, 24). STAT1 might be a molecular marker for the early detection of glioma, as well as a prognostic factor in the determination of glioma aggressiveness (25). Bruton’s tyrosine kinase (BTK) is a nonreceptor tyrosine kinase (26), belonging to the Tec family of kinases (27), expressed in all cell lineages of the hematopoietic system, except for T cells. BTK is a 77 kDa protein essential for B-lymphocyte development, differentiation, and signaling (28) and is currently involved in both physiological and oncogenic pathways through B cell receptor (BCR) regulation (29). Recently, high expression of BTK was associated with glioma tumorigenesis (30) and found to be a potentially novel prognostic marker for poor survival in patients with glioma (31).

We demonstrate the upregulation of CPVL in both glioma cells and tissues. The oncogenic role of CPVL was identified and the STAT1 pathway proved to be the downstream target. CPVL suppressed glioma cell apoptosis by physically interacting with BTK and downregulating the STAT1 phosphorylation through promoting p300-mediated STAT1 acetylation. Thus, our results provide insight into the molecular mechanism underlying the oncogenic role of CPVL in glioma progression. They suggest that CPVL could be a therapeutic target for the treatment of human glioma.

## Results

### CPVL was overexpressed in human glioma cells and tissues.

**Figure 1:**
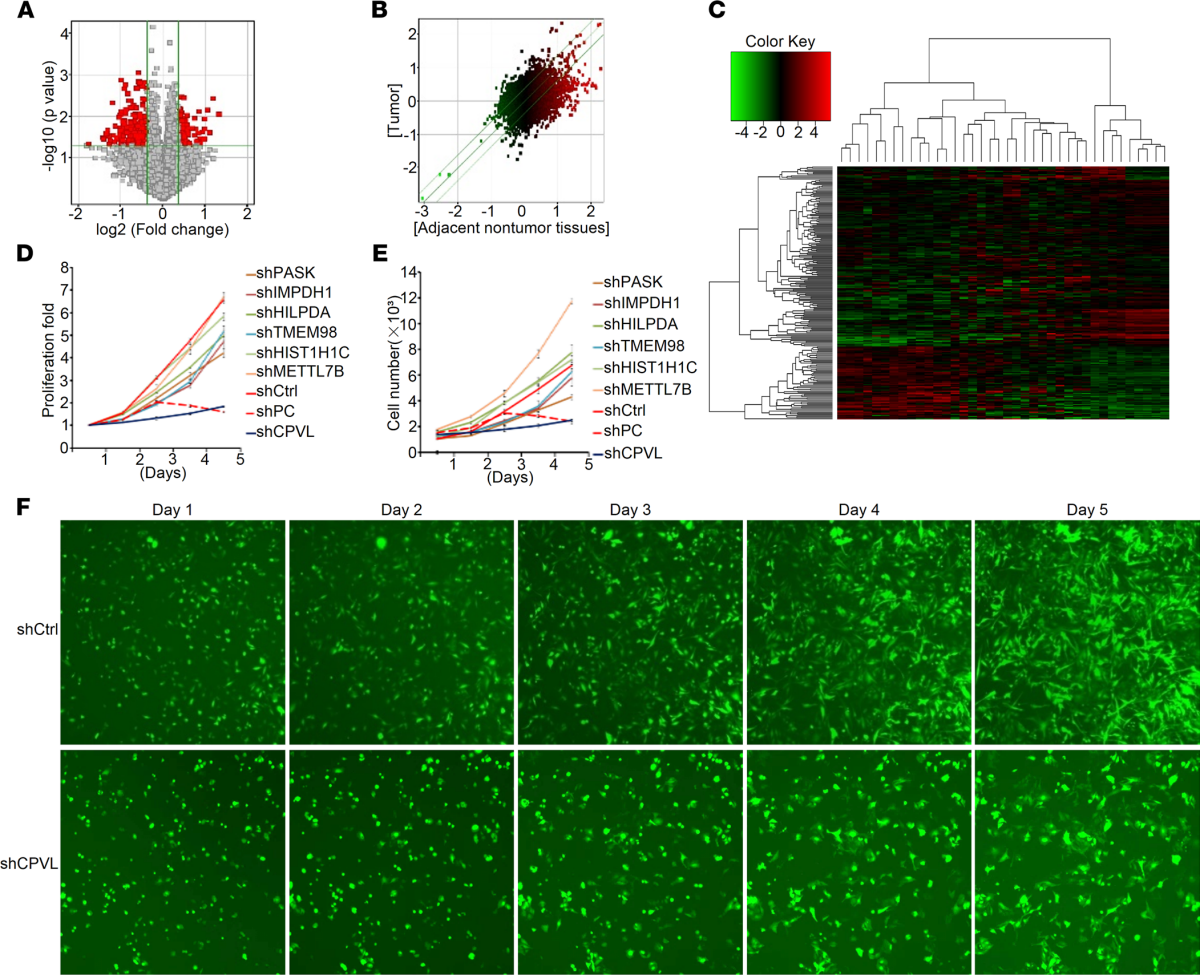
Gene expression profile analysis of CPVL in gliomas. (**A**) Volcanic map of differentially expressed genes in 19 human glioma tissues and corresponding adjacent noncancerous tissues. Red dots are significantly differentially expressed genes, and gray dots are nonsignificantly differentially expressed genes. (**B**) Scatter plot of differentially expressed genes in 19 human glioma tissues and corresponding adjacent noncancerous tissues. The parallel green solid line is the difference reference line, and the points within the reference line represent the probe group with no significant change. Red points outside the reference line represent the probe group with relatively upregulated expression in the glioma tissues group, and green points represent the probe group with relatively upregulated expression in the adjacent noncancerous tissues group. (**C**) Clustering analysis of differentially expressed genes in 19 human glioma tissues and corresponding adjacent noncancerous tissues. Red indicates that the gene expression level is relatively upregulated, green indicates that the gene expression level is relatively downregulated, black indicates that there is no significant change in the gene expression, and gray indicates that the signal intensity of the gene is not detected. (**D**–**F**) The effect of CPVL inhibition on cell proliferation was detected by high-content screening (HCS). The data are shown as mean ± SD. Total original magnification, 200×.

**Figure 2:**
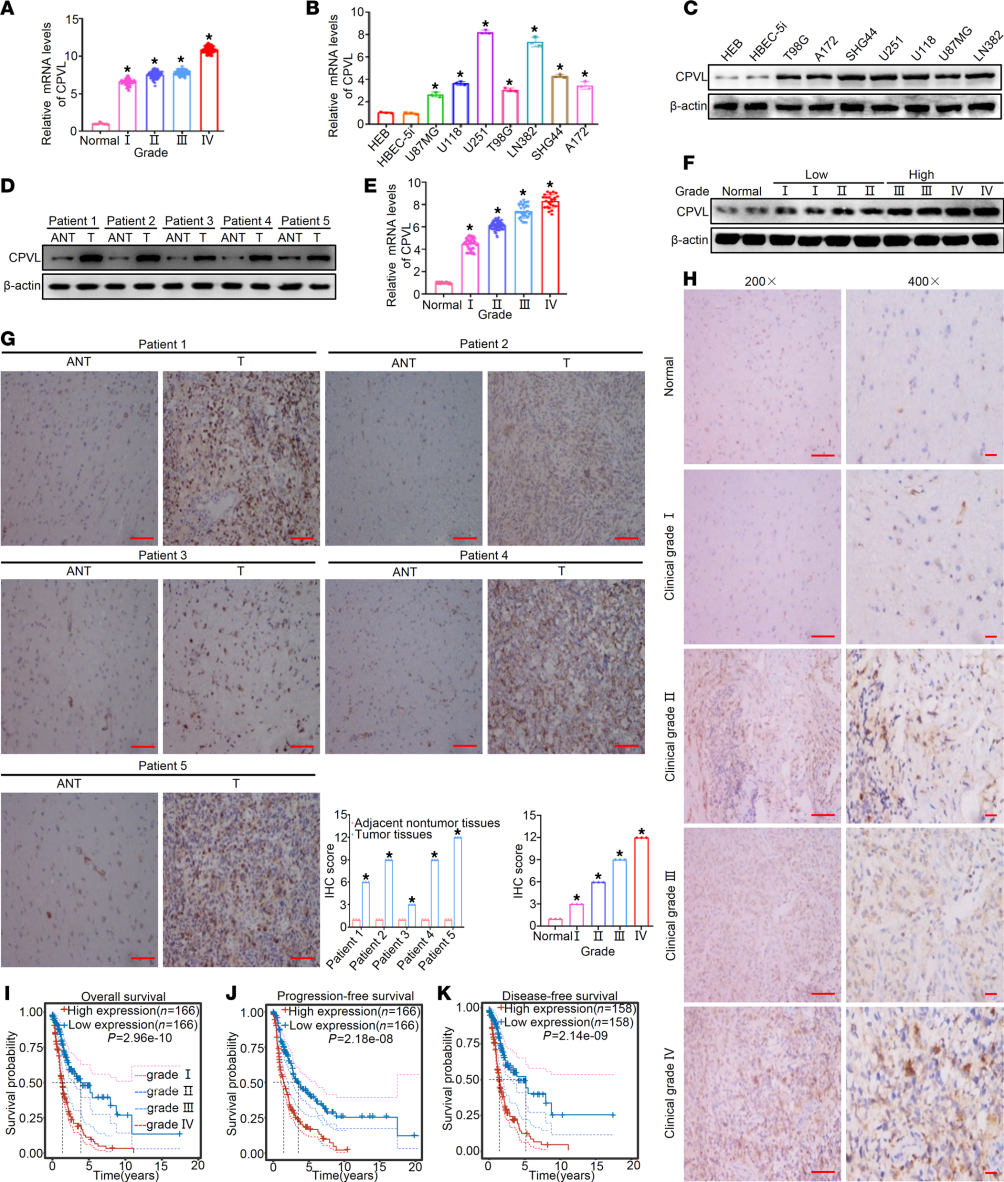
CPVL expression is upregulated in glioma and is associated with poor patient prognoses. (**A**) Relative CPVL mRNA expression in normal brain specimens and glioma specimens of different clinical grades acquired from TCGA (*n* = 10 for normal, *n* = 100 for grade I, *n* =102 for grade II, *n* = 102 for grade III, *n* = 102 for grade IV). (**B** and **C**) The expression level of CPVL in normal brain cell (HEB and HBEC-5i) and glioma cell lines (U251, LN382, SHG44, A172, U118, T98G, and U87MG). (**D**) Western blotting analysis of CPVL expression in matched primary glioma tissues (T) and adjacent noncancerous tissues (ANT). The clinical grades of patientswere characterized (patient 1, grade II; patient 2, grade III; patient 3, grade I; patient 4, grade III; patient 5, grade IV). (**E** and **F**) The expression level of CPVL in normal brain specimens and glioma specimens of different clinical grades(*n* = 20 for normal, *n* = 45 for grade I, *n* = 78 for grade II, *n* = 32 for grade III, *n* = 24 for grade IV). (**G**) IHC staining analysis of CPVL protein expression in matched primary glioma tissues (T) and adjacent noncancerous tissues (ANT). Scale bars: 100 μm (200× magnification). (**H**) IHC staining analysis of CPVL protein expression in normal brain tissues and glioma tissues of different clinical grades. Scale bars: 100 μm (200× magnification, left panels) and 50 μm (400× magnification, right panels). Representative IHC images and IHC score quantification for CPVL are shown. (**I**–**K**) Kaplan-Meier survival curves show overall survival (**I**), progression-free survival (**J**), disease-free survival (**K**) of high CPVL–expressing and low CPVL–expressing glioma patients from the TCGA database. The clinical grades of patients were characterized (patient 1, grade II; patient 2, grade III; patient 3, grade I; patient 4, grade III; patient 5, grade IV).All experiments were repeated 3 times. β-Actin was used as a loading control. Bar graph data are presented as mean ± SD. One-way ANOVA with Dunnett’s multiple comparisons test (**A**,** B**,** E**, and** H**), 2-tailed Student’s *t* test (**G**), and log-rank test (**I**–**K**) analyses were performed. *P < 0.05.

Firstly, we performed microarray expression profiling to analyze the differentially expressed genes between 18 human glioma tissues and corresponding adjacent nontumor tissues. In total, 385 upregulated genes and 764 downregulated genes, such as *HIST1H1C*, *CPVL*, *RFC4*, and *IMPDH1*, were identified (Figure 1, A–C). To further screen relevant functional genes, we used the TCGA database and the Gene chip, which identified 7 candidate genes, *IMPDH1*, *TMEM98*, *HIST1H1C*, *METTL7B*, *PASK*, *CPVL*, and *HILPDA*. We next evaluated these 7 candidate genes through high-content screening (HCS) analysis, which showed that CPVL silencing significantly inhibited the proliferation of glioma cells (Figure 1, D–F). These CPVL silencing data prompted us to examine its expression level in more glioma tissues. The mRNA level of CPVL in 529 glioma tissues was significantly upregulated compared with 10 normal brain tissues from TCGA database (Figure 2A). As CPVL was first characterized in human macrophages, we used the Single cell Glioma RNA-sequence database (TISCH, http://tisch.comp-genomics.org/) to validate that the macrophage cell in glioma is the major source of CPVL (Supplemental Figure 1A; supplemental material available online with this article; https://doi.org/10.1172/jci.insight.146362DS1). What’s more, double-staining with antibodies against macrophage marker (CD68) and CPVL proved that the macrophage cell in glioma contributes to the CPVL expression (Supplemental Figure 1B). Real-time PCR and Western blotting analyses show that CPVL expression was upregulated in 7 glioma cell lines, U87MG, SHG44, LN382, U251, A172, U118, and T98G, compared with the normal glial cell HEB and the human cerebral endothelial cell line HBEC-5i at the transcript and protein levels (Figure 2, B and C). Furthermore, the expression of CPVL in 5 our human glioma tissues was higher than that in matched adjacent noncancerous tissues (Figure 2D), which verified the upregulation of CPVL in glioma.

### CPVL expression predicted the clinical features of glioma.

To validate the mRNA and protein levels of CPVL in glioma tissues of different grades, we conducted quantitative PCR (qPCR) and Western blotting in the low-grade (WHO I/II) and high-grade (WHO III/IV) glioma groups. CPVL expression was higher in high-grade (WHO III/IV) glioma compared with low-grade (WHO I/II) glioma (Figure 2, E and F). To further investigate the relationship between CPVL expression and the clinical features of glioma, CPVL expression was examined in 179 formalin-fixed, paraffin-embedded, archived glioma tissues using IHC staining. The statistical analysis of the IHC staining results are summarized in Supplemental Table 3. IHC staining showed that CPVL expression in glioma tissues was dramatically higher than that in matched adjacent noncancerous tissues (Figure 2G). Moreover, CPVL expression gradually increased, along with advanced clinical grade and poor pathologic differentiation (Figure 2H). Kaplan-Meier survival curves and the log-rank test survival analysis for overall survival (OS), progression-free survival (PFS), and disease-free survival (DFS) in all patients with glioma from the TCGA database showed that the OS, PFS, and DFS of glioma patients with high levels of CPVL were significantly shorter than patients with low levels of CPVL expression (*P* < 0.05; Figure 2, I–K, and Supplemental Tables 4–6). In addition, similar OS results were obtained in WHO IV glioma patients from CGGA database (*P* < 0.05; Supplemental Figure 2C). Similar results were also obtained in another validation cohort of glioma patients from our laboratory (Supplemental Figure 1, D–G). Taken together, the above results suggest that CPVL may be a prognostic factor for survival in patients with glioma.

### CPVL silencing inhibited proliferation and promoted apoptosis of glioma cells in vitro.

**Figure 3:**
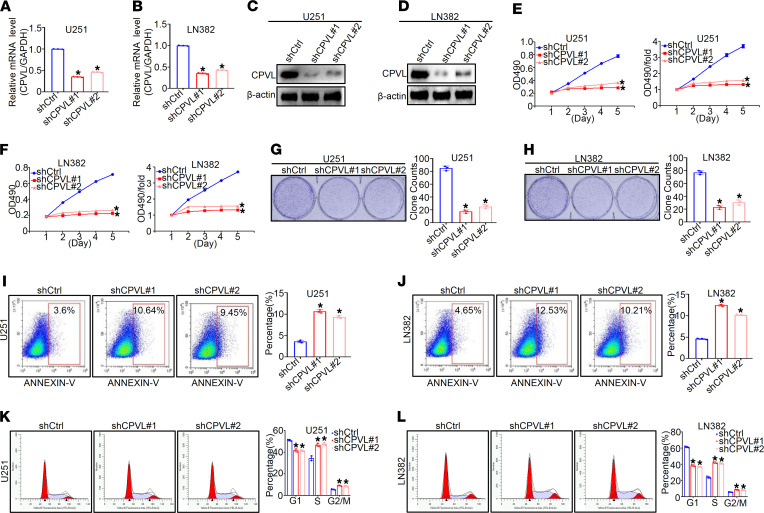
CPVL silencing inhibits the proliferation, promotes apoptosis, and regulates cell cycle of glioma cells in vitro. (**A **and** B**) Relative CPVL mRNA expression in U251 and LN382 cells expressing CPVL shRNA#1 and CPVL shRNA#2 determined by real-time PCR (*n* = 3). (**C **and** D**) Western blotting analysis of CPVL expression in CPVL-silenced U251 and CPVL-silenced LN382 cells (*n* = 3). (**E **and** F**) MTT assays were used to investigate the cell proliferation rates in CPVL-silenced U251 and CPVL-silenced LN382 cells (*n* = 3). (**G **and** H**) Colony formation assay was used to investigate the cell proliferation capacity of the CPVL-silenced U251 and CPVL-silenced LN382 cells. Representative pictures are shown on the left, and the number of colonies counted are shown on the right (*n* = 3). (**I **and** J**) FACS assay was used to detect the effect of cell apoptosis in CPVL-silenced U251 and CPVL-silenced LN382 cells (*n* = 3). Representative profiles are shown on the left, and the percentages of cells that were statistically analyzed are shown on the right. (**K **and** L**) Cell cycle assays were used to investigate the influence of CPVL silencing on cell cycle in CPVL-silenced U251 and CPVL-silenced LN382 cells (*n* = 3). The fractions of viable cells in the G1, S, and G2-M phases were quantified by flow cytometry. Representative profiles are shown on the left, and the percentages of cells that were statistically analyzed are shown on the right. All experiments were repeated 3 times. β-Actin was used as a loading control. Bar graph data are presented as mean ± SD. One-way ANOVA with Dunnett’s multiple comparisons test analyses were performed. *P < 0.05.

To evaluate the function role of CPVL in glioma cells, we stably knocked down the expression of CPVL by 2 CPVL-specific lentiviral shRNAs (shCPVL#1 and shCPVL#2) in U251 and LN382 cells. As shown in Figure 3, A–D, shCPVL#1 exhibited the most evident knockdown effect and, thus, was chosen for the subsequent in vivo and mechanism experiments (*P* < 0.05; Figure 3, A–D). Cell proliferation rate dramatically decreased in CPVL-silenced U251 and LN382 cells compared with the control cells (*P* < 0.05; Figure 3, E and F), which echoed with the results obtained from colony formation assays (*P* < 0.05; Figure 3, G and H). Next, the effects of CPVL expression on cell apoptosis and the cell cycle were further investigated. The percentage of apoptotic cells significantly increased in CPVL-silenced U251 and LN382 cells (*P* < 0.05; Figure 3, I and J). A higher rate of apoptotic cells was also detected by TUNEL staining in CPVL-silenced U251 and LN382 cells (Supplemental Figure 2, A and B). CPVL suppression dramatically decreased the percentage of G1-phase cells and increased the percentage of cells in the S phase and G2/M phase compared with the control cells (*P* < 0.05; Figure 3, K and L). Meanwhile, rescue experiments were conducted by overexpressing CPVL with CPVL cDNA in CPVL-silenced U251 cells and LN382 cells. As expected, CPVL cDNA reversed all of the effects of CPVL silencing on U251 cells and LN382 cells in vitro. Namely, it rescued the diminished rate of cell proliferation, the increased rate of cell apoptosis, the decreased G1 phase, the increased S /G2/M phase, and the decreased tumor growth in vivo (Supplemental Figure 3, A–R). Furthermore, we found that CPVL silencing has no influence on the proliferation and apoptosis of the normal glial cell HEB and the human cerebral endothelial cell line HBEC-5i (Supplemental Figure 4, A–J). In addition, mutagenesis of the CPVL serine carboxypeptidase active site (Δ1[100–400 bp]) was shown to completely abolish the above functions of CPVL (Supplemental Figure 5, A–D). Taken together, these results indicate that CPVL silencing inhibited the proliferation and promoted apoptosis of glioma cells to enhance glioma progression.

### CPVL silencing compromised the tumorigenicity of glioma cells in vivo.

**Figure 4:**
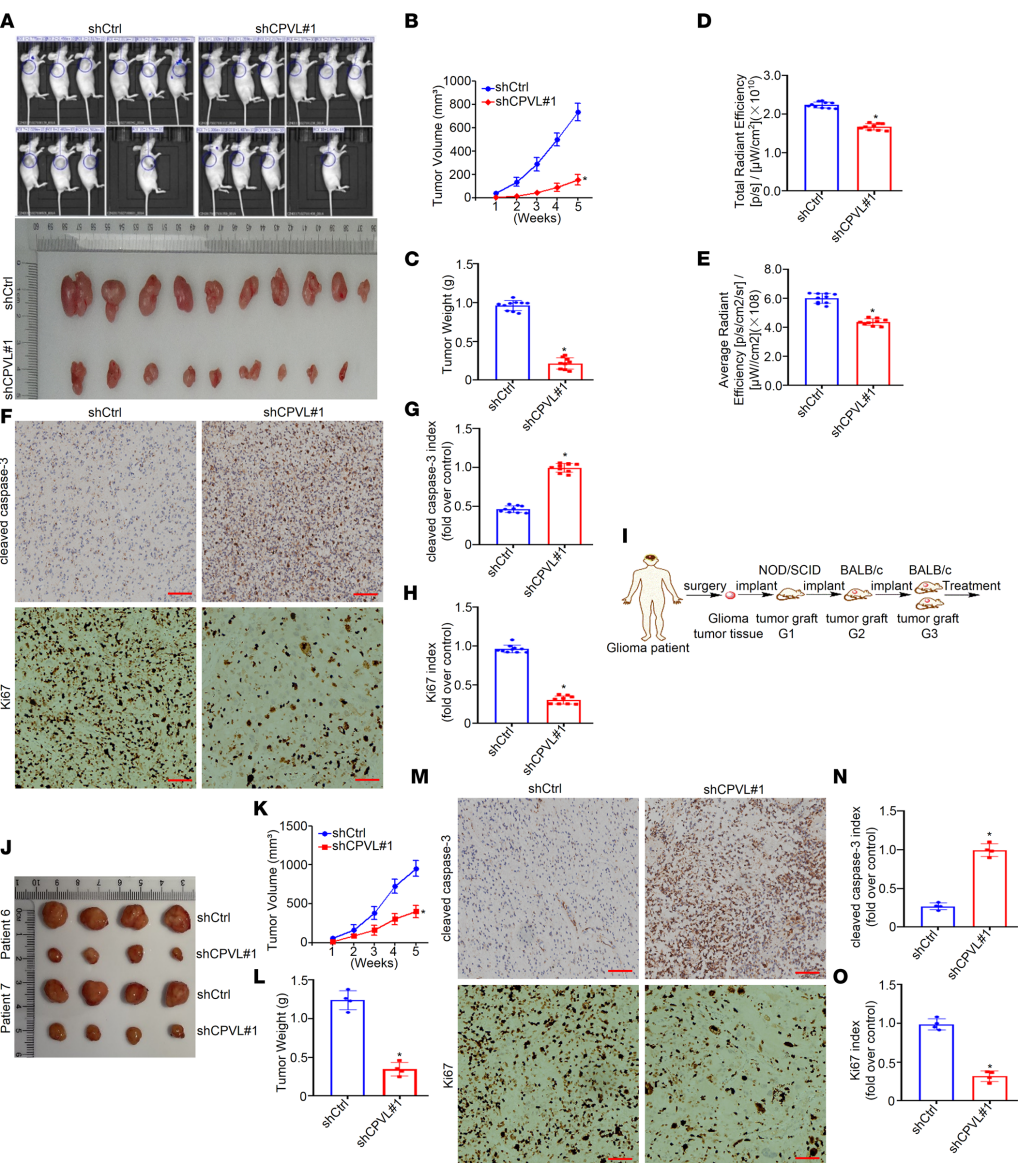
CPVL silencing inhibits the tumorigenicity of glioma cells in vivo. (**A**) Xenograft model in nude mice. The indicated amount of glioma cells was inoculated into the nude mice (*n* = 10/group). Representative images of tumor growth. (**B**) Tumor volume growth curves (*n* = 10). (**C**) Mean tumor weights 28 days after inoculation (*n* = 10). (**D**) Bar graph of the total radiant efficiency of tumor grown from the inoculated glioma cells in BALB/c nude mice (*n* = 10). (**E**) Bar graph of the average radiant efficiency of tumor grown from the inoculated glioma cells in BALB/c nude mice (*n* = 10). (**F**–**H**) The expression levels of cleaved caspase-3 and Ki-67 were determined in xenograft model tumor tissues using IHC (*n* = 10). (**I**) An illustration of the construction of glioma PDX mouse models. (**J**) The engrafted tumors in the shCtrl and shCPVL groups were harvested. The clinical grades of patients were characterized (patient 6, grade III; patient 7, grade IV). (**K**) Tumor volume growth curves of the engrafted tumors were plotted (*n* = 4). (**L**) Tumor weight of the engrafted tumors was recorded (*n* = 4). (**M**–**O**) The expression levels of cleaved caspase-3 and Ki-67 were determined in PDX tumor tissues using IHC (*n* = 4). Scale bars: 100 μm (200× magnification) for panels. All data are presented as the mean ± SD. Two-tailed Student’s *t* test analyses were performed. *P < 0.05.

Stable CPVL-silenced U251 cells were generated, and these cells were inoculated s.c. into the right dorsal flank of BALB/c nude mice. Tumor sizes were monitored every week, and tumor growth curve was then plotted. Tumor-bearing mice and harvested tumors are shown in Figure 4A, which showcased that CPVL inhibition compromised tumor growth dramatically. CPVL inhibition significantly decreased tumor growth rate (*P* < 0.05; Figure 4B). The tumor weight was decreased in the CPVL-silencing group compared with that in the control group (*P* < 0.05; Figure 4C). Quantitative bioluminescence imaging was performed 4 weeks after injection of stable CPVL-silenced or control U251 cells. The total radiant efficiency and average radiant efficiency of CPVL-silenced tumors were weaker than the control tumors at each different group BALB/c nude mice (Figure 4, D and E). We then determined the expression levels of cleaved caspase 3 and Ki-67 in the 2 groups of xenograft mouse tumor models by IHC. As presented, upregulated cleaved caspase 3 and downregulated Ki-67 expression levels were detected in the CPVL-silencing group (*P* < 0.05; Figure 4, F–H). The oncogenic function of CPVL was further confirmed in the patient-derived xenograft (PDX) mouse models of human glioma. We intratumorally injected recombinant shCtrl on one group of PDX mice and shCPVL on another group of PDX mice (Figure 4I). As indicated in Figure 4, J–O, we found that knockdown of CPVL significantly suppressed tumor growth, as illustrated by decreased tumor growth rates, tumor weights, and Ki-67 and increased cleaved caspase 3 indexes from the shCPVL PDX mouse models compared with that of tumors from the control PDX mouse models (*P* < 0.05; Figure 4, J–O). What’s more, we also performed the intracranial PDX glioma models to further validate the oncogenic function of CPVL (Supplemental Figure 6, A–G). These data further support the notion that CPVL silencing inhibited the proliferation and tumorigenicity of glioma cells.

### IFN-γ/STAT1 signaling pathway was identified as downstream target of CPVL.

To elucidate the underlying mechanism by which CPVL contributed to the progression of glioma, microarray analysis was performed to compare the gene expression between CPVL-silenced U251 and control cells. Differentially expressed genes with at least a 1.5-fold change were identified. We found 320 significantly upregulated genes and 366 significantly downregulated genes with CPVL knockdown (Supplemental Figure 7A and Supplemental Figure 8, A and B). Next, we used the Database for Annotation Bioinformatics Microarray Analysis (www.ingenuity.com) to determine whether particular gene sets were significantly enriched in these differentially expressed genes regulated by CPVL. Forty-two functional classifications, as annotated by Ingenuity Pathway Analysis (IPA; www.ingenuity.com), were enriched, including Interferon Signaling, Antigen Presentation Pathway, and Estrogen-mediated S-phase Entry. Interferon Signaling was significantly activated (Supplemental Figure 7B). Then, the IPA software was used to predict the activation of the upstream regulators. When the activation *Z* score algorithm was used, the successful prediction of the upstream regulators by random was significantly reduced. In this study, IFN-γ/STAT1 was predicted to be strongly activated, and 108 uniformly activated genes were identified (Supplemental Figure 7C and Supplemental Table 7). Disease and functional analysis based on the IPA software demonstrated that cell apoptosis was significantly enriched (Supplemental Figure 7D and Supplemental Figure 8, C and D). Analysis of regulatory effects based on IPA software found that CPVL was involved in the regulation of apoptosis of glioma cells through the IFN-γ/STAT1 signaling pathway (Supplemental Figure 7E). Supplemental Figure 7F intuitively showed expression changes in genes related to the IFN-γ/STAT1 signaling pathway, providing the basis for studying the molecular mechanism involved in the observed changes between groups (Supplemental Figure 7F).

### CPVL inhibition induced glioma cell apoptosis via the STAT1 signaling pathway.

**Figure 5:**
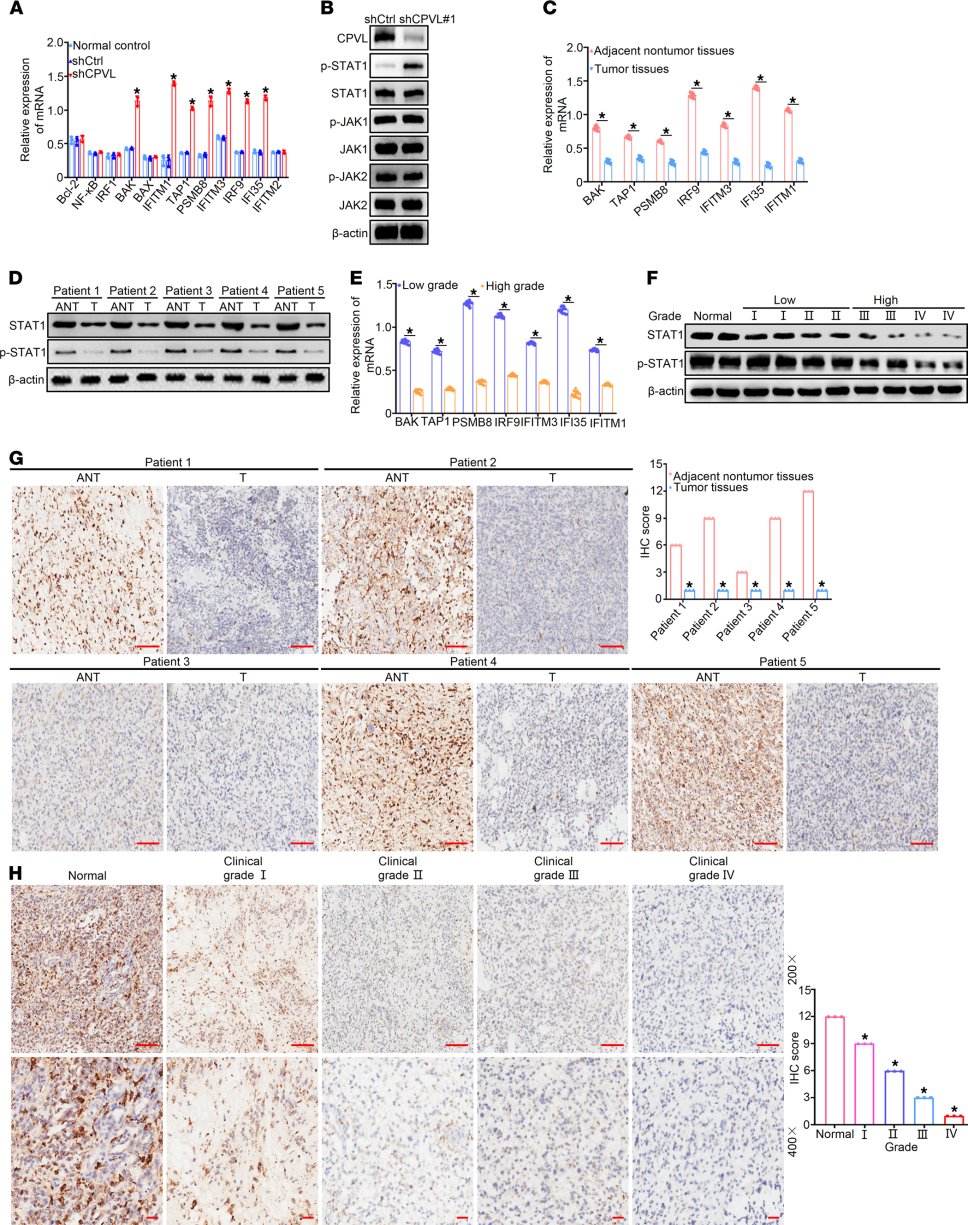
CPVL induces apoptosis of glioma cells via STAT1 signaling pathway. (**A**) Relative IFN-γ/STAT1 signaling pathway downstream response gene mRNA expression in CPVL-silenced U251 cells determined by real-time PCR (*n* = 3). (**B**) Western blotting analysis of the IFN-γ/STAT1 signaling pathway downstream response genes’ protein expression in CPVL-silenced U251 cells. (**C **and **D**) Relative IFN-γ/STAT1 signaling pathway downstream response gene mRNA expression in matched primary glioma tissues (T, *n* = 60) and adjacent noncancerous tissues (ANT, *n* = 60). (**E **and **F**) The expression level of relative the IFN-γ/STAT1 signaling pathway downstream response genes in glioma specimens of low clinical grades and high clinical grades. (**G**) IHC staining analysis of the expression of p-STAT1 in matched primary cancer tissues (T) and adjacent noncancerous tissues (ANT). Representative IHC images (left) and IHC score quantification (right) for CPVL in tissue sections are shown. Scale bars: 100 μm (200× magnification). (**H**) IHC staining analysis of the expression of p-STAT1 in normal brain tissues and glioma tissues of different clinical grades. Representative IHC images (left) and IHC score quantification (right) for CPVL in tissue sections are shown. Scale bars: 100 μm (200× magnification, upper panels) and 50 μm (400× magnification, lower panels). The clinical grades of patients were characterized (patient 1, grade II; patient 2, grade III; patient 3, grade I; patient 4, grade III; patient 5, grade IV). All experiments were repeated 3 times. β-Actin was used as a loading control. Bar graph data are presented as mean ± SD. One-way ANOVA with Dunnett’s multiple comparisons test (**H**), and 2-tailed Student’s *t* test (**A**,** C**,** E**, and **G**) analyses were performed. *P < 0.05.

To verify changes in the expression of IFN-γ/STAT1 signaling pathway downstream response genes in the function of CPVL, qPCR was conducted to compare the STAT1 signaling pathway downstream response genes expression of CPVL-silenced glioma U251 cells with control cells. Similar to what is shown in Supplemental Figure 7F, the downstream response genes IRF9, IFI35, PSMB8, IFITM1, IFITM3, TAP1, and BAK were significantly activated after CPVL knockdown (Figure 5A). Furthermore, we examined the protein expression of the genes related to the STAT1 signaling pathway of CPVL-silenced U251 cells compared with the control cells by Western blotting. The results revealed that the phosphorylation levels of STAT1 significantly increased upon CPVL inhibition, whereas that of JAK1, JAK2, p-JAK1, and p-JAK2 did not change (Figure 5B). To further verify the relative levels of the STAT1 signaling pathway downstream response genes in adjacent noncancerous tissues/primary cancer tissues (ANT/T) paired glioma, real-time PCR and Western blotting analyses were performed, which showed that the mRNA and protein expression levels of the STAT1 signaling pathway downstream response genes in 5 pairs of human glioma tissues were lower than those in matched adjacent noncancerous tissues (Figure 5, C and D, and Supplemental Figure 9A). To further validate the mRNA and protein expression levels of the STAT1 signaling pathway downstream response genes in glioma tissues of different grades, qPCR and Western blotting of low-grade (WHO I/II) and high-grade (WHO III/IV) glioma groups were performed. The results showed higher expression of the STAT1 signaling pathway downstream response genes in the low-grade (WHO I/II) glioma group compared with the high-grade (WHO III/IV) glioma group (Figure 5, E and F, and Supplemental Figure 9B). To further investigate the relationship between STAT1 signaling pathway downstream response gene expression and the clinical features of glioma, STAT1 signaling pathway downstream response gene expression was examined in formalin-fixed, paraffin-embedded, archived glioma tissues using IHC staining. The results reveal that the expression levels of STAT1 signaling pathway downstream response genes in glioma tissues were lower than those in matched adjacent noncancerous tissues and further decreased with advanced clinical grade and in the tumors with poor pathologic differentiation (Figure 5, G and H; Supplemental Figure 10, A–D; Supplemental Figure 11, A–D; and Supplemental Figure 12, A–D). These results suggest that CPVL may promote glioma progression via inhibiting the STAT1 signaling pathway.

**Figure 6:**
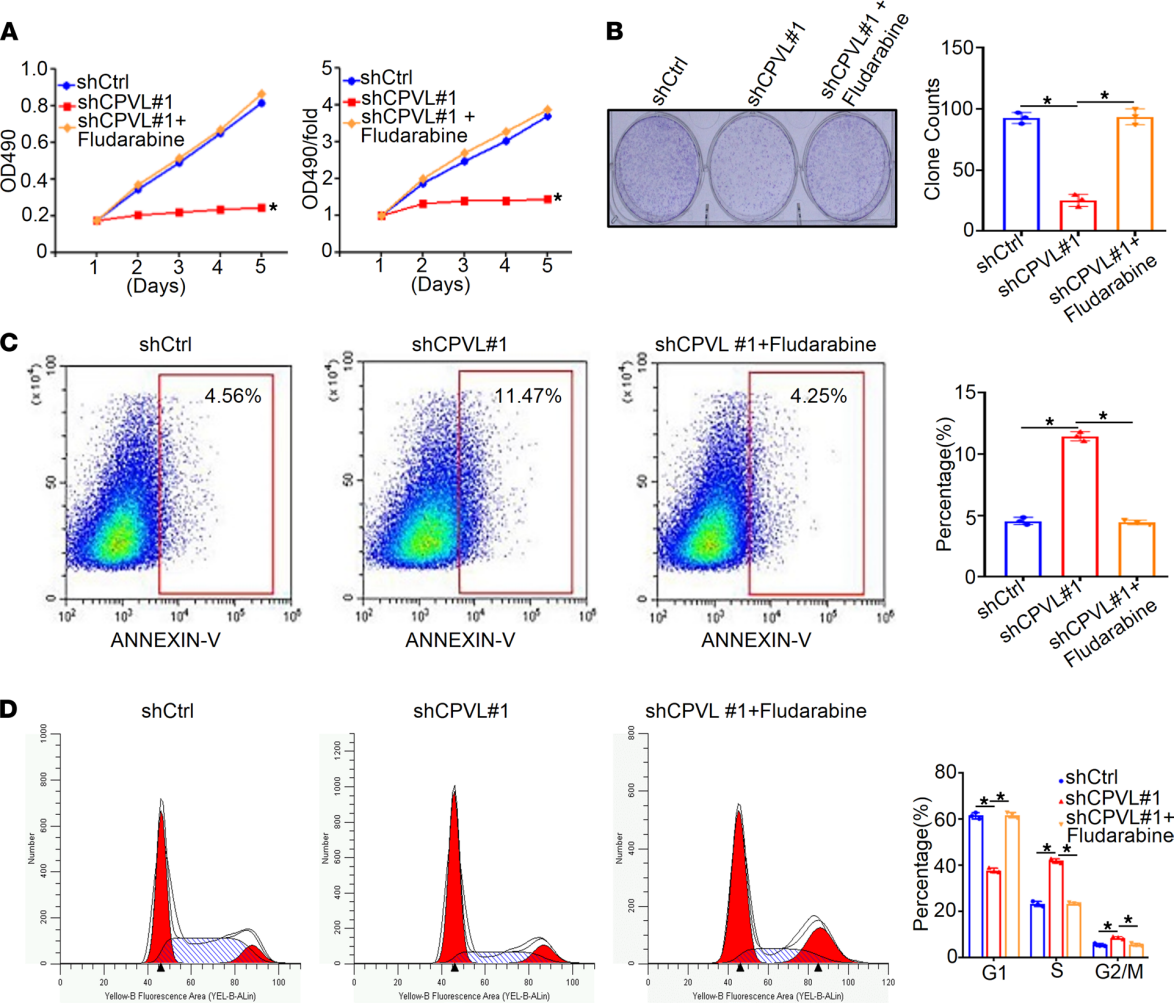
Rescue experiments verify that CPVL regulates apoptosis of glioma cells via the IFN-γ/STAT1 signaling pathway. (**A**) MTT assays were used to investigate the cell proliferation rates of CPVL-silenced U251 cells treated with fludarabine compared with the control cells (*n* = 3). (**B**) Colony formation assay was used to investigate the cell proliferation capacity of CPVL-silenced U251 cells treated with fludarabine, compared with the control cells. Representative pictures are shown on the left, and the number of colonies counted is shown on the right (*n* = 3). (**C**) FACS assay was used to detect the effect of fludarabine treatment on cell apoptosis in CPVL-silenced U251 cells, compared with the control cells (*n* = 3). Representative profiles are shown on the left, and the percentages of cells that were statistically analyzed are shown on the right. (**D**) Cell cycle assays were used to investigate the influence of fludarabine treatment on the CPVL-silenced U251 cell cycle compared with the control cells (*n* = 3). The fractions of viable cells in the G1, S, and G2-M phases were quantified by flow cytometry. Representative profiles were shown on the left, and the percentages of cells that were statistically analyzed are shown on the right. All experiments were conducted in triplicate. Bar graph data are presented as the mean ± SD. Two-tailed Student’s *t* test analyses were performed.*P < 0.05.

We then further employed rescue experiments to verify the above results. We demonstrated that phospho-STAT1 inhibitor fludarabine could rescue the diminished cell proliferation rate of CPVL-silenced U251 cells. These results were further confirmed by colony formation assays (Figure 6, A and B). Furthermore, flow cytometry indicated that fludarabine attenuated the increased percentage of apoptotic cells upon CPVL inhibition (Figure 6C) and dramatically recovered the percentage of G1-phase and G2/M-phase cells induced by CPVL knockdown (Figure 6D). Taken together, these results show that CPVL silencing induced glioma cell apoptosis via activating STAT1 signaling pathway.

### CPVL physically interacted with BTK to downregulate the STAT1 phosphorylation through promoting p300-mediated STAT1 acetylation.

**Figure 7:**
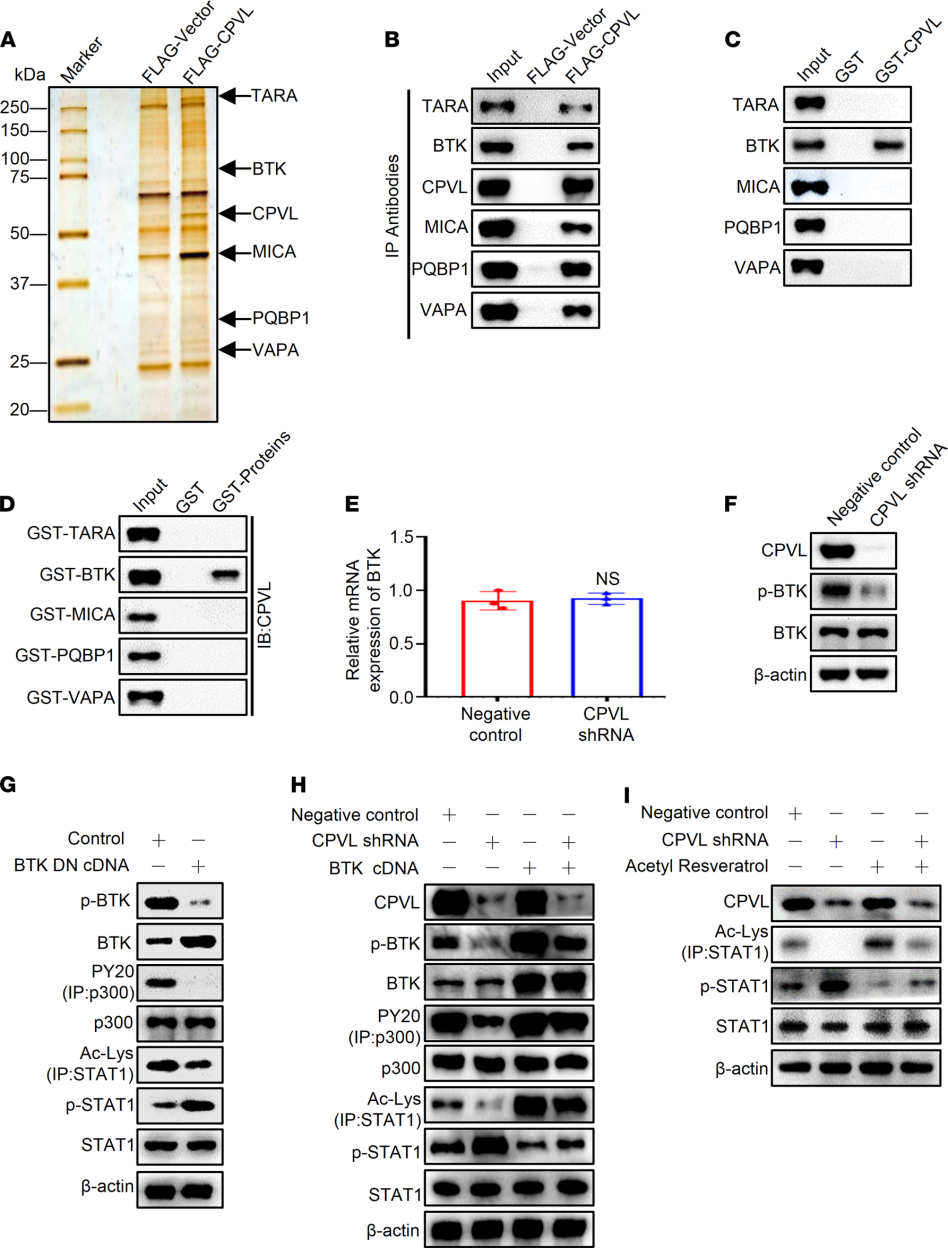
CPVL physically interacts with BTK to regulate the STAT1 phosphorylation through p300-mediated STAT1 acetylation. (**A**) Immunoaffinity purification of CPVL-containing protein complex. Cellular extracts from U251 cells stably expressing FLAG vector or FLAG-CPVL were immunopurified with anti-FLAG affinity columns and eluted with FLAG peptide. These eluates were resolved by SDS-PAGE and were silver stained. (**B**) IP of whole-cell lysates from U251 cells followed by IB with antibodies against the indicated proteins. (**C**) GST pull-down assays with GST-fused CPVL and in vitro transcribed/translated TARA, BTK, MICA, PQBP1, or VAPA as indicated. (**D**) GST pull-down assays with the indicated GST-fused proteins and in vitro transcribed/translated CPVL. (**E **and **F**) U251 cells were transfected with a control shRNA or CPVL shRNA. The mRNA level of BTK and the protein levels of CPVL, BTK, and p-BTK were measured. (**G**) U251 cells were treated with control cDNA or BTK DN cDNA, and the protein levels of p-BTK, BTK, p300, PY20 (IP: p300), Ac-Lys (IP: STAT1), p-STAT1, and STAT1 were measured by Western blotting. (**H**) Rescue experiments were used to investigate whether the biological function of CPVL was mediated by regulating phosphorylation of BTK. U251 cells were transfected with CPVL shRNA or CPVL shRNA plus BTK cDNA compared with the control cells, and the protein levels of CPVL, p-BTK, BTK, p300, PY20 (IP: p300), Ac-Lys (IP: STAT1), p-STAT1, and STAT1 were measured by Western blotting. (**I**) Rescue experiments were used to investigate the STAT1 phosphorylation in CPVL-silenced U251 cells treated with acetylation activator, acetyl resveratrol, compared with the control cells, and the protein levels of CPVL, STAT1, Ac-Lys (IP: STAT1), and p-STAT1 were measured by Western blotting. All experiments were repeated 3 times. β-Actin was used as a loading control. Bar graph data are presented as mean ± SD. Two-tailed Student’s *t* test analyses were performed.

In order to clarify the underlying mechanisms of CPVL that regulated the STAT1 signaling pathway, we performed affinity purification and mass spectrometry (MS) to identify proteins physically associated with CPVL. The lysates of U251 cells expressing FLAG-CPVL were prepared and subjected to FLAG affinity purification. The eluates were resolved on SDS-PAGE and were silver stained. The results of MS analysis indicate that CPVL was combined with a number of proteins, including TARA, BTK, MICA, PQBP1, and VAPA (Figure 7A). Detailed results are provided in Supplemental Table 8. Each of 6 antibodies against the 6 indicated proteins was used to immunoprecipitate their corresponding proteins from U251 cells transfected with FLAG-vector or FLAG-CPVL; then, the CPVL antibody was used to examine whether CPVL was coimmunoprecipitated with each of the 6 proteins. IP with anti-FLAG antibody, followed by immunoblotting (IB) with antibodies against the above proteins, showed that they were efficiently coimmunoprecipitated with CPVL (Figure 7B). To further support the physical association between CPVL and TARA, BTK, MICA, PQBP1, or VAPA, GST pull-down experiments were performed. Incubation of GST-fused CPVL with in vitro transcribed/translated TARA, BTK, MFGN, PQBP1, or VAPA revealed that CPVL interacted directly with BTK (Figure 7C). Reciprocal GST pull-down experiments with GST-fused TARA, BTK, MFGN, PQBP1, or VAPA and in vitro transcribed/translated CPVL yielded similar results (Figure 7D). Collectively, these results indicate that CPVL was physically associated with BTK.

BTK is a nonreceptor tyrosine kinase that belongs to Tec family (18). In this study, we clarified the effects of CPVL on BTK, and the data show that downregulation of CPVL did not affect the mRNA or total protein levels of BTK (Figure 7, E and F). Interestingly, the phosphorylation level of BTK was decreased in CPVL-silenced U251 cells (Figure 7F). These results reveal that CPVL affected the phosphorylation level of BTK. Previous studies have revealed that BTK via tyrosine phosphorylation and activation of 300 kDa protein (p300) facilitate STAT1 acetylation, which is followed by antagonism of STAT1 phosphorylation (32–34). In order to verify this conclusion, U251 cells were transfected with dominant negative BTK cDNA (BTK DN cDNA), which potently inhibited BTK autophosphorylation on Tyr223. As presented in Figure 7G, reduced BTK phosphorylation led to decreased p300 phosphorylation, which weakened STAT1 acetylation and increased STAT1 phosphorylation (Figure 7G). To further investigate whether the biological function of CPVL was mediated by the phosphorylation of BTK, U251 cells were transfected with CPVL shRNA or CPVL shRNA plus BTK cDNA. As shown in Figure 7H, CPVL silencing significantly inactivated phosphorylation of BTK, which decreased p300-mediated STAT1 acetylation and increased STAT1 phosphorylation, whereas the effects of CPVL shRNA were weakened following the introduction of BTK cDNA (Figure 7H). As expected, BTK cDNA could rescue the diminished cell proliferation rate, the increased apoptotic cells of CPVL-silenced U251 cells (Supplemental Figure 13, A–C). To further validate CPVL-mediated STAT1 phosphorylation that was caused by STAT1 acetylation, we performed the rescue experiments. The results indicate that acetylation activator acetyl resveratrol can attenuate the diminished STAT1 acetylation level and the increased STAT1 phosphorylation level in CPVL-silenced U251 cells (Figure 7I). Meanwhile, acetyl resveratrol could attenuate the diminished cell proliferation rate and the increased apoptotic cells of CPVL-silenced U251 cells (Supplemental Figure 13, D–F). Taken together, these experiments show that CPVL directly interacted with BTK to downregulate the STAT1 phosphorylation through promoting p300-mediated STAT1 acetylation.

## Discussion

Tumorigenesis is a complex multistep process characterized by uncontrolled cell growth, and tumor formation is largely associated with progressive accumulation of genetic and epigenetic alterations in genes or proteins that regulate cell proliferation (35). Therefore, identification of genes and proteins that lead to glioma tumorigenesis is critical for the establishment of new diagnostic and prognostic methods (36). The key findings of our present study are the oncogenic role of CPVL, and CPVL silencing inhibited the cell proliferation and tumorigenicity of glioma. CPVL is located at the 7p14–p15 region in human chromosome (16, 37). Chromosome 7 is amplified in glioma, and this may contribute to CPVL overexpression in glioma (38), suggesting that it is possibly an oncogene. However, Wilms tumors have been linked to deletions or translocations around chromosome 7p15, the region containing the CPVL gene, suggesting that CPVL may act as a tumour suppressor gene (39). Hu et al. found that abnormal CPVL expression is associated with retinopathy in Chinese patients with type 2 diabetes (40). Ran et al. found that CPVL is highly expressed in gastric cancer tissues, but the correlation between CPVL and gastric cancer was not further investigated (41). The clinical significance and biological role of CPVL in glioma progression, however, remains largely unknown. Here, we found that CPVL was significantly upregulated in a large cohort of human glioma tissues and that CPVL expression levels were significantly correlated with the clinical characteristics of glioma, including clinical grades. Importantly, survival analysis showed that patients with low expression of CPVL had better OS compared with those with high levels of CPVL, suggesting that CPVL is a predictor for survival of patients with glioma.

Expression of CPVL was originally identified in human macrophages (16), and CPVL is strongly expressed in human alveolar macrophages in vivo (42). CPVL can be modulated by inflammatory stimuli. The protein is downregulated in macrophages cultured in medium with IFN-γ and *Staphylococcus aureus* (43). The function of this enzyme remains unclear. CPVL as a true serine carboxypeptidase remains unconfirmed, although the primary sequence displays the expected serine carboxypeptidase active site, and our attempts to detect protease activity in the supernatants of 293T cells transfected with CPVL were unsuccessful, although this may be due to the fact that these cells do not process CPVL in the appropriate manner into a mature form. The primary sequence of CPVL contains a putative signal peptide sequence, 4 potential N-linked glycosylation sites, and 4 myristoylation sites but no transmembrane domain, suggesting that it may be a luminal protein in an organelle and/or involved in the secretory pathway (43). In this study, we found that CPVL silencing promoted glioma sensitivity to cell apoptosis. We also demonstrated that downregulating CPVL with CPVL-specific lentiviral shRNAs may be a novel strategy for the treatment of glioma. Increased resistance to apoptosis is a hallmark alteration in most types of cancers (44). The abrogation of proapoptotic pathways has been demonstrated to be one of the key events during tumor development and progression, and impairment of apoptotic programming is tightly linked to the commonly observed failure of anticancer chemotherapy and radiotherapy (45). Thus, the elucidation of the mechanisms modulating the apoptosis/survival process in a particular cancer type may generate novel insights in developing more effective therapeutic strategies (46). Notably, in the current study, we found that CPVL plays an important role in antiapoptosis of glioma that is relatively insensitive to chemotherapy, both in vivo and in vitro. Suppressing CPVL expression in glioma cells dramatically enhanced glioma cell apoptosis, suggesting that CPVL activity contributes to sustaining the unwanted survival of glioma cells under the treatment of chemotherapeutics. Thus, the key functional gene CPVL in this context, which contributes to determining whether a cell undergoes proliferation or apoptosis, could be a potential target for novel anticancer drugs.

To address the hypothesis that CPVL plays critical roles in promoting proliferation and tumorigenicity, inhibiting apoptosis, and affecting other possible cellular processes required for glioma development, microarray and differential expression profiling in relation to CPVL were performed. Interestingly, a large number of genes that were differentially regulated following CPVL knockdown were significantly linked to cellular apoptosis processes. IPA revealed that the IFN-γ/STAT1 signaling pathway is a downstream target pathway affected by CPVL that results in glioma cell apoptosis. IFN-γ is a cytokine with important roles in tissue homeostasis, immune and inflammatory responses, and tumor immunosurveillance (47). Signaling by the IFN-γ receptor activates the JAK/STAT1 pathway to induce the expression of classical IFN-stimulated genes that have key immune effector functions (48). An increasing amount of evidence indicates that inhibition of the IFN-γ/STAT1 signaling pathway may prove to be a viable therapeutic target for a subset of malignancies (49). Previous studies have demonstrated that the IFN-γ/STAT1 signaling pathway plays important roles in glioma apoptosis (50). A prominent mechanism linking the IFN-γ/STAT1 signaling pathway to cancer progression is the abrogation of apoptosis (51). STAT transcription factors are generally activated in cancer, and STAT1 phosphorylation acts as a tumor suppressor by inducing cell apoptosis and reduced cell proliferation (20, 21). The present study has determined that CPVL silencing can significantly activate the IFN-γ/STAT1 signaling pathway, resulting in the apoptosis of glioma cells. Regarding the mechanism involved, we demonstrate that CPVL directly interacts with BTK to downregulate the STAT1 phosphorylation through promoting p300-mediated STAT1 acetylation. BTK is a TEC-family nonreceptor tyrosine kinase that signals downstream of numerous cellular receptors, including the BCR, TLR, and Fc receptors (FcR) (32). BTK functions as a promising target in solid tumors, including glioma, and its degradation acts as a therapeutic strategy for cancer (31, 52). Importantly, the clinically approved covalent BTK inhibitor ibrutinib has been approved for use in cancer patients. What’s more, ibrutinib is a promising therapeutic strategy for glioma, blocking the glioma cell proliferation, migration, and invasion properties and inducing glioma cell apoptosis and autophagy, targeting the Akt/mTOR pathway (53). Previous studies indicate that BTK is a dual-function regulator of apoptosis (54). In this study, we demonstrate that CPVL is physically associated with BTK. The previous studies have revealed that activation of BTK causes p300-mediated STAT1 acetylation, which in turn antagonizes STAT1 phosphorylation (33, 34). The transcriptional coactivator p300 plays a role in histone acetylation and chromatin remodeling, and an increase in p300 activity has been linked to a more aggressive phenotype in several cancers (55). Consistent with previous results, our experimental results indicate that the BTK/p300 axis is critically involved in the STAT1 acetylation. Collectively, our study demonstrates that CPVL directly interacts with BTK to downregulate the STAT1 phosphorylation through promoting p300-mediated STAT1 acetylation, supporting the pursuit of CPVL as a potential target for glioma intervention.

In addition, several other issues also remain to be addressed. For example, our studies were performed in low-passage glioma cell lines that may not represent the comprehensive biology of GBM in vivo. Therefore, these results obtained from these low-passage glioma cell lines, ideally, need to be validated in primary cultures of GBM. We did not carry out these valiations, and this is a limitation of our studies. In addition, it would be of great interest to know whether other pathways and types of glioma cells could also be modulated by upregulating CPVL. These issues are currently being investigated in our laboratory. Nevertheless, understanding the role of CPVL in glioma progression will improve our understanding of the mechanisms underlying glioma survival, as well as help establish CPVL as a potential therapeutic target for the treatment of glioma.

In summary, our results suggest that, as a novel functional gene, CPVL plays a pivotal role in human glioma progression. Full understanding of the precise role of CPVL in human glioma may provide the opportunity to develop a novel therapeutic strategy by suppressing its expression in glioma cells. In addition, CPVL may be utilized as a clinical indicator of disease progression and as a prognostic marker for patient survival. Translational research on the clinical use of CPVL is required to generate a methodology and evaluate the molecular diagnostic applications of CPVL in glioma.

## Methods

### Cell lines

The human normal glial cell line HEB and 4 human glioma cell lines (U87MG, U251, A172, and T98G) were obtained from the American Type Culture Collection (ATCC). The human cerebral endothelial cell line HBEC-5i and 3 glioma cell lines (SHG44, LN382, and U118) were purchased from the China Academia Sinica Cell Repository (Shanghai, China). The cell lines were characterized by DNA fingerprinting, cell vitality detection, isozyme detection, and mycoplasma detection. The last cell characterization was performed in December, 2020. All cell lines were routinely cultured at 37°C in a 5% CO_2_ humidified atmosphere in DMEM (Thermo Fisher Scientific) with 10% FBS (Thermo Fisher Scientific).

### Lentivirus production and cell transfection

The lentiviral vector system was purchased from Genechem Technology (Shanghai, China). Full-length CPVL was cloned into the H145 pLenti-EF1a-EGFP-F2A-Puro-CMV-MCS vector. A lentiviral vector encoding the human CPVL was designated as CPVL cDNA, and an empty plasmid vector served as a control vector (pcDNA 3.1). Lentiviral constructs containing the shRNA sequence targeting human CPVL were inserted into the pLKD-CMV-G&PR-U6-shRNA vector and were used to establish cell lines constitutively repressing CPVL (named shCPVL#1 and shCPVL#2). For lentiviral shRNA transfection, U251 and LN382 cells were seeded in 6-well plates (1 × 10^5^ cells/well) and transfected with negative control (NC) shRNA or CPVL-specific lentiviral shRNA, or/and BTK-specific lentiviral shRNA (Genechem Co. Ltd.) when the cell density reached 30%–50% confluence according to the manufacturer’s instructions. For plasmid transfection, U251 and LN382 cells were seeded in 6-well plates (1 × 10^5^ cells/well) and transfected with control vector plasmid or CPVL cDNA, BTK cDNA, or BTK DN cDNA (Genechem Co. Ltd.) when the cell density reached 70%–80% confluence, according to the manufacturer’s instructions. Glioma cells were infected with concentrated virus in the presence of polybrene (8 μg/mL; Sigma-Aldrich) and were then selected with puromycin (8 μg/mL).

### Tissue samples

In this study, tumor tissues were obtained from 179 glioma patients, and nonneoplastic brain samples were obtained from traumatic brain injury patients at the Department of Neurosurgery of the First Affiliated Hospital of Wannan Medical College (Wuhu, Anhui, China) from February 2015 to October 2018. Two pathologists evaluated all specimens according to the 2016 WHO guidelines. No local or systemic treatments were administered to these patients before surgery. The tissues were immediately frozen in liquid nitrogen and stored at –80°C until use. All of the patients provided signed, informed consent before the use of these clinical materials for research purposes. The use of these archival tissues in this study was approved by the Ethics Committee of the First Affiliated Hospital of Wannan Medical College. The clinical information of the samples is summarized in Supplemental Table 1.

### Differentially expressed genes analysis

Total RNA was isolated from 19 human glioma tissues and corresponding adjacent nontumor tissues using TRIzol reagent kit (Thermo Fisher Scientific), and RNA integrity was assessed by Agilent Bioanalyzer 2100 (Agilent Technologies). The samples were subjected to hybridization and scanned on the Affymetrix Human Gene Expression Array (Affymetrix) according to the manufacturer’s instructions. RNA-Seq profiling data are available via Gene Expression Omnibus (GEO) GSE188256. Differentially expressed genes data analyses, including the Cancer Genome Atlas (TCGA) Data Portal (https://portal.gdc.cancer.gov/), the Chinese Glioma Genome Atlas (CGGA) data portal (http://www.cgga.org.cn/index.jsp), and the GEO database (https://www.ncbi.nlm.nih.gov/geo/), were performed using R software.

### HCS

HCS includes 3 steps. Step 1 is fell infection; step 2 is high content image acquisition and analysis; and step 3 is screening the positive genes related to cell proliferation.

#### Cell infection.

Glioma cells were seeded in a 96-well black-bottom plate to achieve a density of 1500–2500 cells/well. Twenty-four hours later, cells were infected with different specific lentiviral shRNAs (Genechem Co. Ltd.) as recommended by the manufacturer. When the 70%–90% cells were expressed GFP under fluorescence microscopy, cells were used in the following experiments.

#### High content image acquisition and analysis.

The infected cells were seeded in 96‑well plates to achieve a density of 2 × 10^3^ cells in 100 μL per well and cultured at 37°C with 5% CO_2_. Each group had 3 wells. Twenty-four hours later, the GFP image of glioma cells was captured continuously using the Celigo cell cytometer (Nexcelom Bioscience) for 5 days. The number of glioma cells with GFP was counted by the software.

#### Screening the positive genes related to cell proliferation.

According to the cell count and time point, the cell growth curve was plotted. Compared with the first day, the cell count ratio at each time point of each group was calculated. According to the ratio and time point, the curve of cell growth ratio was plotted. Compared with the NC group, the fold change of cell growth ratio of each group at fifth day was calculated. Fold change (NC/experimental group) = cell growth ratio of NC group/cell growth ratio of experimental group. When fold change ≥ 2, the target genes in the experimental group were positive genes related to cell proliferation.

### qPCR analysis

Total RNA was extracted from the transfected cells with TRIzol (Invitrogen), and 0.4 μg RNA was used to synthesize cDNA using a first-strand cDNA synthesis kit (Thermo Fisher Scientific). The RNA concentration was examined using a NanoDrop 2000 spectrophotometer (Thermo Fisher Scientific). qPCR analysis was performed using the CFX-96 (Bio-Rad) according to the manufacturer’s instructions. Data were normalized according to the level of GAPDH expression in each sample. GAPDH was used as internal control, and 2^–*ΔΔ*Ct^ values were used to assess the relative expression of the target gene. Primers were provided by RiBoBio (Guangzhou) as described in Supplemental Table 2. Each sample was repeated in triplicate and analyzed using the Relative Quantification Software.

### IHC and tissue microarray

IHC analysis of 179 paraffin-embedded glioma patient tumor tissue sections was performed and diagnosed by 2 pathologists blinded to patient identity. Briefly, the sections were deparaffinized with xylenes and rehydrated in an ethanol series. Sections were submerged in EDTA antigenic retrieval buffer (pH 8) and microwaved for antigenic retrieval. The sections were then treated with 3% hydrogen peroxide in methanol to quench the endogenous peroxidase activity, followed by incubation with 1% goat serum albumin to block nonspecific binding. The tissue sections were incubated with the corresponding antibody overnight at 4°C. After washing, the tissue sections were treated with goat anti–mouse/rabbit IgG HRP-polymer (catalog sc-2005/sc-2004, Santa Cruz Biotechnology Inc.) for 20 minutes. 3, 3’-Diaminobenzidine was used as the chromogen. The tissue microarrays (TMAs) for 179 paraffin-embedded glioma tissue sections were constructed following the manufacturer’s instructions. Briefly, tissue cylinders with a diameter of 0.6 mm were taken from the selected regions of donor block and then punched precisely into a recipient paraffin block using a tissue arraying instrument (Beecher Instruments). Consecutive, 5 μm sections of the microarray blocks were made with a microtome. The H-scores were determined by combining the intensity of staining and the proportion of positively stained tumor cells. The intensity was graded as follows: 0, negative; 1, weak; 2, moderate; 3, strong. The proportion of positive tumor cells was graded: 0, 0%–5%; 1, 5%–25%; 2, 26%–50%; 3, 51%–75%; 4, 75%–100%. A final score was derived by the multiplication of the 2 primary scores. Final scores of 0–4 were defined as negative. The final scores of 0–6 were defined as low expression and scores of 8–12 as a high expression to analyze the correlation between CPVL expression and glioma.

### Western blotting

Protein extract after various treatments was electrophoresed, separated by 10% SDS-PAGE, and transferred onto a 0.22 μm nitrocellulose (NC) membrane (GE Healthcare). The NC membrane was blocked in 5% nonfat milk at room temperature for 1 hour and then probed with the an indicated primary antibody overnight at 4°C. Antibodies (1:1000) against anti-STAT1 (catalog 14994), anti–phospho-STAT1 (catalog 9167), anti-JAK1 (catalog 3344), anti–phospho-JAK1 (catalog 74129), anti-JAK2 (catalog 3230), anti–phospho-JAK2 (catalog 3771), anti-BTK (catalog 8547), anti–phospho-BTK (catalog 87141), anti–acetylated-Lysine (catalog 9441), and anti-p300 (catalog 86377) were all obtained from Cell Signaling Technology. Anti-CPVL (Abcam, catalog ab180147), anti-MICA (Abcam, catalog ab222098), anti-PQBP1 (Abcam, catalog ab100797), anti-VAPA (Abcam, catalog ab96584), and anti-phsophotyrosine (PY20) (Abcam, catalog ab10321) were used. Anti-TARA (catalog sc-377474) was bought from Santa Cruz Biotechnology Inc. Anti–β-actin (catalog A1978) was procured from Sigma-Aldrich. The NC membranes were extensively washed 3 times and then incubated with anti–mouse or anti–rabbit horseradish peroxidase–conjugated secondary antibody (catalog 7076S/7074S, Cell Signaling Technology). Following removal of the secondary antibody, the membranes were scanned by Tanon 5200 (Tanon). The band intensity was measured by densitometry using the Quantity One Software (Tanon). The protein levels were normalized with that of β-actin. All experiments were repeated in triplicate, and the representative results were shown.

### Cell apoptosis assay and TUNEL staining

The Annexin V-APC assay was performed to evaluate cell apoptosis with Annexin V-APC Apoptosis Detection Kit (Invitrogen). Briefly, cells were harvested, washed twice with cold PBS, centrifuged at 189*g* for 5 minutes and washed with D-Hanks (pH 7.2–7.4). Binding buffer (1×) was used to resuspend cells, and Annexin V-APC was added to the cells and incubated for 15 minutes at room temperature in the dark. Samples were run on a FACScan ﬂow cytometer (Beckman Coulter), and the percentages of apoptotic cells were analyzed using FlowJo software (Beckman Coulter). TUNEL staining of apoptotic cells was detected in glioma cells using a One Step TUNEL Apoptosis Assay Kit (Beyotime Institute of Biotechnology) following the manufacturer’s protocol. In brief, cells fixed on coverslips with 4% paraformaldehyde for 30 minutes at room temperature and then were treated with 0.1% Triton X-100 for 5 minutes at room temperature. The cells were washed with PBS twice and incubated with 50 μL TUNEL reaction mixture at 37°C for 1 hour. The TUNEL staining cell images were captured using a fluorescence microscope (Zeiss LSM800 confocal microscope). Three independent experiments were performed.

### Cell cycle distribution

The PI-FACS assay was performed to evaluate cell cycle distribution with PI-FACS Cell cycle Detection Kit (MilliporeSigma). In brief, cells were trypsinized, fixed in 70% ethanol, washed once with PBS, and then labeled with propidium iodide in the presence of RNase A for 30 minutes in the dark. Samples were run on a FACScan ﬂow cytometer (Beckman Coulter), and the percentages of cells within each phase of the cell cycle were analyzed using FlowJo software. Three independent experiments were performed.

### Colony formation assay

For the colony formation assay, the cells were plated in 6-well plates at 1 × 10^3^ cells per well and maintained in DMEM containing 10% FBS for 2 weeks. After 2 weeks, the cells were washed 2 times with PBS, fixed with methanol, and stained with crystal violet at the end of the time course prior to the capture of the representative images via camera. The number of colonies was counted under a microscope (Nikon Corporation). All experiments were performed in triplicate.

### MTT assay

Cells were seeded in 96-well plates at initial density of 2 × 10^3^ per well. At each time point, cells were stained with 100 mL sterile MTT dye (0.5 mg/mL, MilliporeSigma) for 4 hours at 37°C, followed by removal of the culture medium and the addition of 100 mL of dimethyl sulfoxide (MilliporeSigma). The absorbance was measured using an Enzyme analyzer (Tecan infinite M2009PR) at 490 nm, with 570 nm as the reference wavelength. All experiments were conducted in triplicate.

### Site-specific mutagenesis of CPVL

CPVL cDNA was amplified using total reverse transcribed cDNA as the template. The amplified PCR fragments were digested with KpnI/EcoRI restriction enzymes and inserted into the pcDNA3.1(+) vector. To construct different length of CPVL, serine carboxypeptidase fragments (Δ1[1–400 bp], Δ2[400–800 bp], Δ3[800–1200 bp], Δ4[1200–1600 bp], Δ5[1600–2021 bp]) were amplified from U251 cells’ genomic DNA by PCR and were then cloned into pGL3-Basic Vector (Promega) at the Kpn I and XhoI sites. Point mutations in the CPVL was generated by site-specific mutagenesis using the overlap PCR extension method.

### Xenografted tumor model

Four- to 6-week-old (average weight, 20 g) BALB/c nude mice (male/female ratio 1:1) were obtained from the Shanghai Institute of Materia Medica, Chinese Academy of Science (Shanghai, China) and maintained under specific pathogen–free conditions. No statistical method was applied for the sample size estimation for the animal study, although to ensure the precision of the results, each experimental group had enrolled 10 nude mice in an unrandomized manner. The experimental protocol was approved by the Wannan Medical College Animal Experimental Ethics Committee. The cells (2 × 10^6^ U251 shCPVL cells and U251 shCtrl cells) were injected s.c. into the right dorsal flank, and tumor sizes were measured using a Vernier caliper every day when the tumors were readily visualized. Tumor formation in nude mice was observed by measuring the tumor volume calculated with the following formula: volume = (length × width^2^)/2. Bioluminescence images were acquired with the IVIS Imaging System (PerkinElmer). Analysis was performed with Living Image software (PerkinElmer) by measuring photon flux of chest and upper abdominal region. On day 28, animals were euthanized and tumors were excised and weighed. The exclusion criteria of these animal experiments are that the body weight of the mouse was statistically significantly changed compared with the others and that the xenograft tumor was festered seriously and influenced the measurement of tumor volume.

### PDX mouse model

NOD/SCID and BALB/c mice were obtained from the Shanghai Institute of Materia Medica, Chinese Academy of Science, and were used for the establishment of the glioma PDX model. Briefly, we collected the primary glioma tissues from 2 patients after surgical resection and kept the specimens in ice-cold culture medium supplemented with 1% penicillin/streptomycin (Thermo Fisher Scientific). Then, the tissues were diced into 2–3 mm^3^ pieces and s.c. implanted into the flanks of NOD/SCID mice. When the xenografted tumors grew up to 1–2 cm^3^, we harvested the tissues from the mice bearing PDX tumors and cut the tissues into pieces. The tumor fragments were further implanted into BALB/c nude mice for the serial transplantation. When the tumor volume reached 50 mm^3^, we intratumorally injected recombinant lentivirus vectors (negative control lentiviral shRNA/CPVL-specific lentiviral shRNA) into tumor tissues continuously for 20 days. Tumor weight and volume were recorded.

### Intracranial PDX mouse model

Single-cell suspensions were generated from 9 patient tumor samples and transfected with negative control shRNA or CPVL-specific lentiviral shRNA when the cell density reached 30%–50% confluence or transfected with an empty vector, or with CPVL overexpression (CPVL cDNA) when the cell density reached 70%–80% confluence, according to the manufacturer’s instructions prior to intracranial implantation. For intracranial implantation, mice were anesthetized before a 1 cm incision was made in the midline of the mouse head to expose the skull underneath. A transcranial burr hole was created using sterile hand-held drill and a mouse was mounted on a stereotaxic device. Glioma cells (2 × 10^4^ cells) in 2.5 μL of sterile PBS were loaded into a 29G Hamilton Syringe and injected slowly over a period of 3 minutes into the left hemisphere of the mouse brain at 3 mm depth through the transcranial burr hole created 3 mm lateral and 2 mm caudal relative to midline and bregma sutures. Following injection, the incision was closed using 9 mm stainless steel wound clips.

### Microarray analysis

Total RNA was isolated from 3 replicate samples of U251-shCtrl and U251-shCPVL cells using TRIzol reagent kit (Thermo Fisher Scientific), and RNA integrity was assessed by Agilent Bioanalyzer 2100 (Agilent Technologies). The samples were subjected to hybridization on the Affymetrix Human Gene Expression Array (Affymetrix) according to the manufacturer’s instructions. Data analyses — including the canonical pathways — and network analyses were performed using the IPA. Profiling data are available via GEO GSE188256.

### Rescue experiments

The rescue experiment of STAT1 inhibitor fludarabine (S1491, Selleck Chemicals) for silencing CPVL in U251 cells was carried out. U215 cells were first stably transfected with NC shRNA or CPVL shRNA, and they were then used Nifuroxazide (20 μM) to inhibit the expression of STAT1 in the CPVL-silenced U215 cells, which were divided into 3 groups, including NC shRNA, CPVL shRNA, and CPVL shRNA/Nifuroxazide groups. The following assays were performed: MTT assay and colony formation assay for cell proliferation and ﬂow cytometry assay for evaluation of cell cycle and cell apoptosis. Three independent experiments were performed. The rescue experiment of acetylation activator acetyl resveratrol (S3934, Selleck Chemicals) for silenced CPVL in U251 cells was carried out. U215 cells were first stably transfected with NC shRNA or CPVL shRNA, which were divided into 4 groups, including NC shRNA, CPVL shRNA, acetyl resveratrol, and CPVL shRNA plus acetyl resveratrol groups. Then protein bands were detected with specific antibodies by Western blotting.

### Immunopurification and MS

U251 cells stably expressing FLAG-CPVL were generated by transfection of the cells with FLAG-tagged CPVL and selection in medium containing 1 mg/mL of G418. Anti-FLAG immunoaffinity columns were prepared using anti-FLAG M2 affinity gel (Sigma-Aldrich) following the manufacturer’s suggestions. Cell lysate was obtained from approximately 5 × 10^8^ cells and applied to an equilibrated FLAG column of 1 mL bed volume to allow for adsorption of the protein complexes to the column resin. After binding, the column was washed with cold BC500 buffer containing 50 mM Tris, 2 mM EDTA, 500 mM KCl, 10% glycerol, and protease inhibitors. FLAG peptide (0.2 mg/mL, Sigma-Aldrich) was applied to the column to elute the FLAG protein complex as described by the vendor. Fractions of the bed volume were collected and resolved on SDS polyacrylamide gels; they were silver stained and subjected to liquid chromatography–tandem MS (LC-MS/MS) sequencing and data analysis.

### Glutathione S-transferase pull-down experiments

Glutathione S-transferase fusion constructs were expressed in *E. coli* BL21 cells, and crude bacterial lysates were prepared by sonication in ice-cold PBS in the presence of a protease inhibitor mixture. The in vitro transcription and translation experiments were performed with rabbit reticulocyte lysate (TNT Systems; Promega). In GST pull-down assays, about 10 μg of the appropriate GST fusion protein was mixed with 5–8 μL of the in vitro transcribed/translated products and incubated in binding buffer (0.8% BSA in PBS in the presence of the protease inhibitor mixture). The binding reaction was then added to 30 μL of glutathione-Sepharose beads and mixed at 4°C for 2 hours. The beads were washed 5 times with binding buffer, resuspended in 30 μL of 2 × SDS-PAGE loading buffer, and resolved on 12% gels. Protein bands were detected with specific antibodies by western blotting.

### Statistics

Statistical analyses were performed with SPSS 13.0 Statistical Software (SPSS Inc.). The difference between 2 or multiple groups was compared with 2-tailed Student’s *t* test or 1-way ANOVA test. Data are presented as the mean ± SD from triplicated independent experiments. The cumulative OS, PFS, and DFS rates were calculated using the Kaplan-Meier method, and differences between curves were evaluated using the log-rank test. A 2-tailed *P* value less than 0.05 was considered to indicate a statistically significant result.

### Study approval

All protocols were approved by the Ethical Scientific Committee of Wannan Medical College (Wuhu, Anhui Province, China).

## Author contributions

KL, MM, and X Liang designed the study. HY wrote the paper. X Liu and XZ performed the cellular, molecular experiments. HY, M Zhang, X Li, and M Zhong performed the other all experiments. TC and LJ analyzed the data. M Zhang and M Zhong contributed reagents/materials and animal housekeeping. All authors approved the final version of the manuscript.

## Supplementary Material

Supplemental data

Supplemental table 1

Supplemental table 2

Supplemental table 3

Supplemental table 4

Supplemental table 5

Supplemental table 6

Supplemental table 7

Supplemental table 8

## Version 1 11/16/2021

In-Press Preview

## Version 2 12/22/2021

Electronic publication
