# Supplementary material for: CPVL promotes glioma progression via STAT1 pathway inhibition through interactions with the BTK/p300 axis
Source: JCI Insight. 2021 Dec 22;6(24):e146362. doi: 10.1172/jci.insight.146362 (PMC8783677; doi:10.1172/jci.insight.146362)
Supplement: Supplemental data [file jciinsight-6-146362-s154.pdf]

Supplemental information

Supplemental figure and figure legends

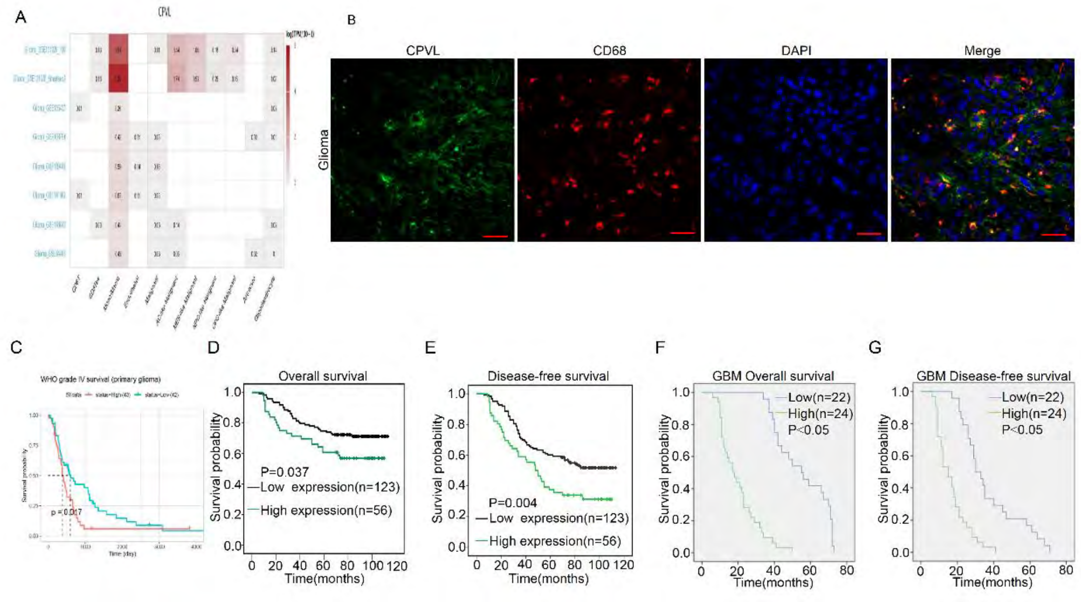

Supplemental Figure 1. CPVL was overexpressed in human glioma cells and tissues.

(**A**) Analysis of the major source of CPVL in glioma from the Single cell Glioma RNA-sequence databases. (**B**) Immunofluorescence analysis of the predominantly localization of macrophage marker (CD68) and CPVL in glioma tissues. The nuclei were stained with DAPI. Scale bars: 50 μm (400× magnification) for panels. (**C**) Kaplan-Meier survival curves show Overall survival of high CPVL-expressing and low CPVL-expressing GBM patients from the CCGA database. (**D**) Kaplan-Meier survival curves show Overall survival (OS) of high CPVL-expressing (n=56) and low CPVL-expressing (n=123) glioma patients from our laboratory. (**E**) Kaplan-Meier survival curves show Disease-free survival (DFS) of high CPVL-expressing (n=56) and low CPVL-expressing (n=123) glioma

patients from our laboratory. (**F**) Kaplan-Meier survival curves show Overall survival (OS) of high CPVL-expressing (n=24) and low CPVL-expressing (n=22) GBM patients from our laboratory. (**G**) Kaplan-Meier survival curves show Disease-free survival (DFS) of high CPVL-expressing (n=24) and low CPVL-expressing (n=22) GBM patients from our laboratory. Log-rank test (**C-G**) analyses were performed.*, $P<0.05$.

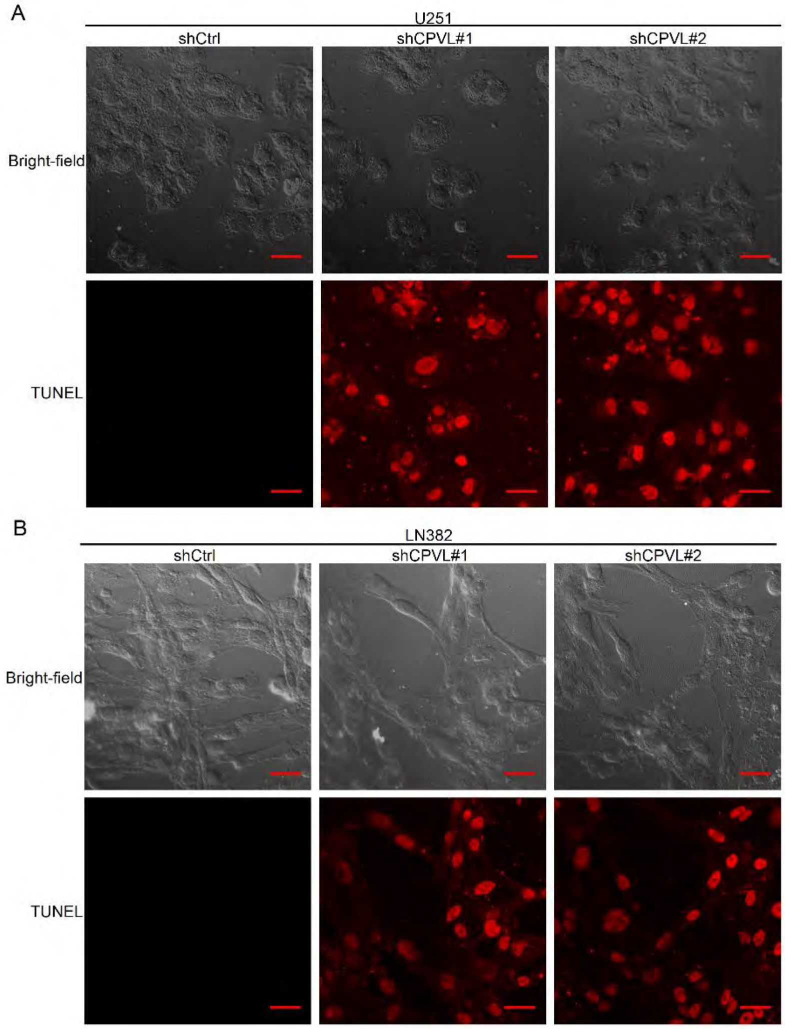

Supplemental Figure 2. CPVL silencing promoted apoptosis of glioma cells *in vitro*.

(**A**) TUNEL staining of apoptotic cells was detected in CPVL-silenced U251 cells. The TUNEL staining cell images were captured using a fluorescence microscope. (**B**) TUNEL staining of apoptotic cells was detected in CPVL-silenced LN382 cells. The TUNEL staining cell images were captured using a fluorescence microscope. Scale bars: 50 µm (400× magnification) for panels.

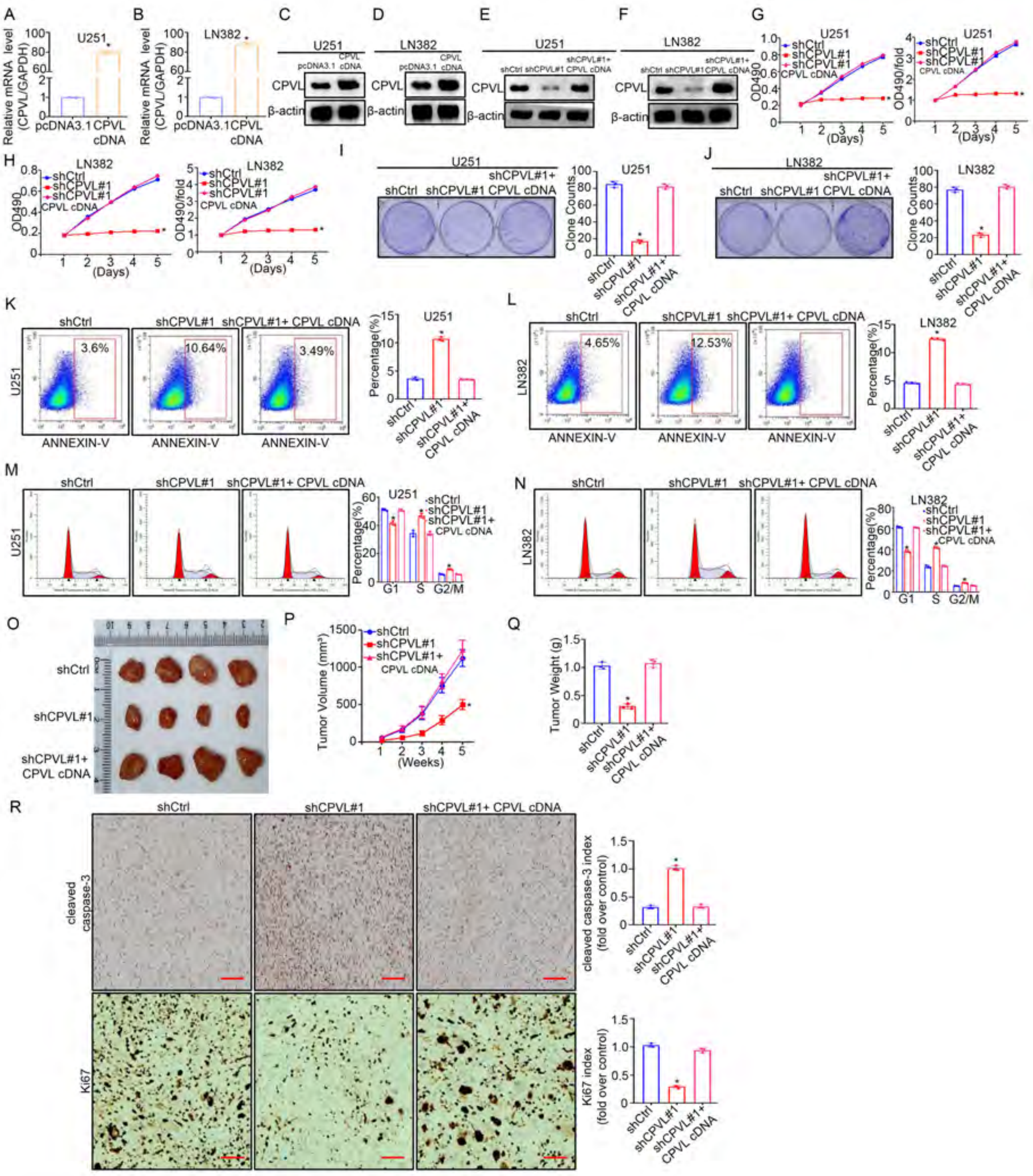

Supplemental Figure 3. Rescue experiments were conducted by overexpressing CPVL with CPVL cDNA in CPVL-silenced U251 cells and LN382 cells.

(**A** and **B**) Relative CPVL mRNA expression in CPVL-overexpressed U251 and CPVL-overexpressed LN382 cells determined by real-time PCR (n=3). (**C** and **D**) Western blotting analysis of CPVL expression in CPVL-overexpressed U251 and CPVL-overexpressed LN382 cells (n=3). (**E** and **F**) Western blotting analysis of CPVL expression in CPVL-silenced, CPVL-silenced plus CPVL-overexpressed U251 and LN382 cells (n=3). (**G** and **H**)MTT assays were used to investigate the proliferation rates in CPVL-overexpressed treatment on CPVL-silenced U251 and CPVL-silenced LN382 cells compared to the control cells (n=3). (**I** and **J**) Colony formation assay was used to investigate the proliferation capacity of the CPVL-overexpressed treatment on CPVL-silenced U251 and CPVL-silenced LN382 cells compared to the control cells (n=3). Representative pictures are shown on the left, and the number of colonies was counted on the right. (**K** and **L**) FACS assay was used to detect the effect of cell apoptosis in CPVL-overexpressed treatment on CPVL-silenced U251 and CPVL-silenced LN382 cells compared to the control cells (n=3). Representative profiles were shown on the left and the percentage of cells was statistically analyzed on the right. (**M** and **N**) Cell cycle assays were used to investigate the influence of CPVL-overexpressed treatment on

CPVL-silenced U251 and CPVL-silenced LN382 cells compared to the control cells (n=3). The fractions of viable cells in the G1, S, and G2–M phases were quantified by flow cytometry. Representative profiles are shown on the left and the percentage of cells was statistically analyzed on the right. (**O**) Xenograft model in nude mice. Indicated cells were inoculated into the nude mice (n=4). Representative images of tumor growth. (**P**) Tumor volume growth curves (n=4). (**Q**) Mean tumor weights 28 days after inoculation (n=4). (**R**) The expression levels of cleaved caspase-3 and Ki67 were determined in xenograft model tumor tissues using immunohistochemistry (n=4). All experiments were repeated three times in triplicate. β-actin was used as loading control. Bar graph data are presented as mean ± SD. Two-tailed Student's test analyses were performed. *, $P<0.05$.

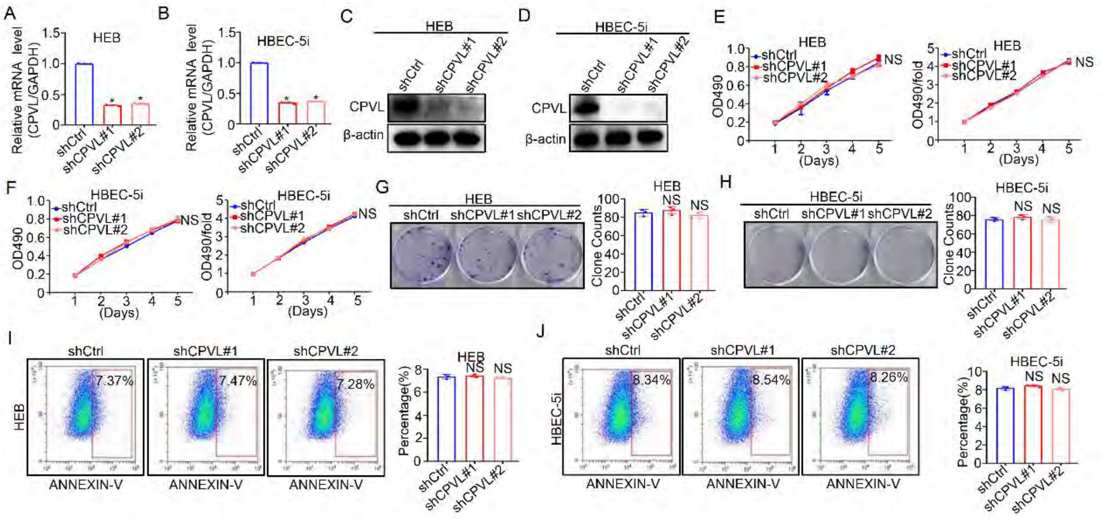

Supplemental Figure 4. CPVL silencing has no influence on the proliferation and apoptosis of the normal glial cell HEB and the human cerebral endothelial cell line HBEC-5i.

(**A** and **B**) Relative CPVL mRNA expression in HEB and HBEC-5i cells expressing CPVL shRNA#1 and CPVL shRNA#2 determined by real-time PCR (n=3). (**C** and **D**) Western blotting analysis of CPVL expression in CPVL-silenced HEB and CPVL-silenced HBEC-5i cells (n=3). (**E** and **F**) MTT assays were used to investigate the proliferation rates in CPVL-silenced HEB and CPVL-silenced HBEC-5i cells (n=3). (**G** and **H**) Colony formation assay was used to investigate the proliferation capacity of the CPVL-silenced HEB and CPVL-silenced HBEC-5i cells (n=3). Representative pictures are shown on the left, and the number of colonies was counted on the right. (**I** and **J**) FACS assay was used to detect the effect of cell apoptosis in CPVL-silenced HEB and CPVL-silenced HBEC-5i cells (n=3). Representative profiles were shown on the left and the percentage of cells was statistically analyzed on the right. All experiments were repeated three times in triplicate. β-actin was used as loading control. Bar graph data are presented as mean ± SD. One-way ANOVA with Dunnett's multiple comparisons test analyses were performed. *, $P<0.05$. NS, $P>0.05$.

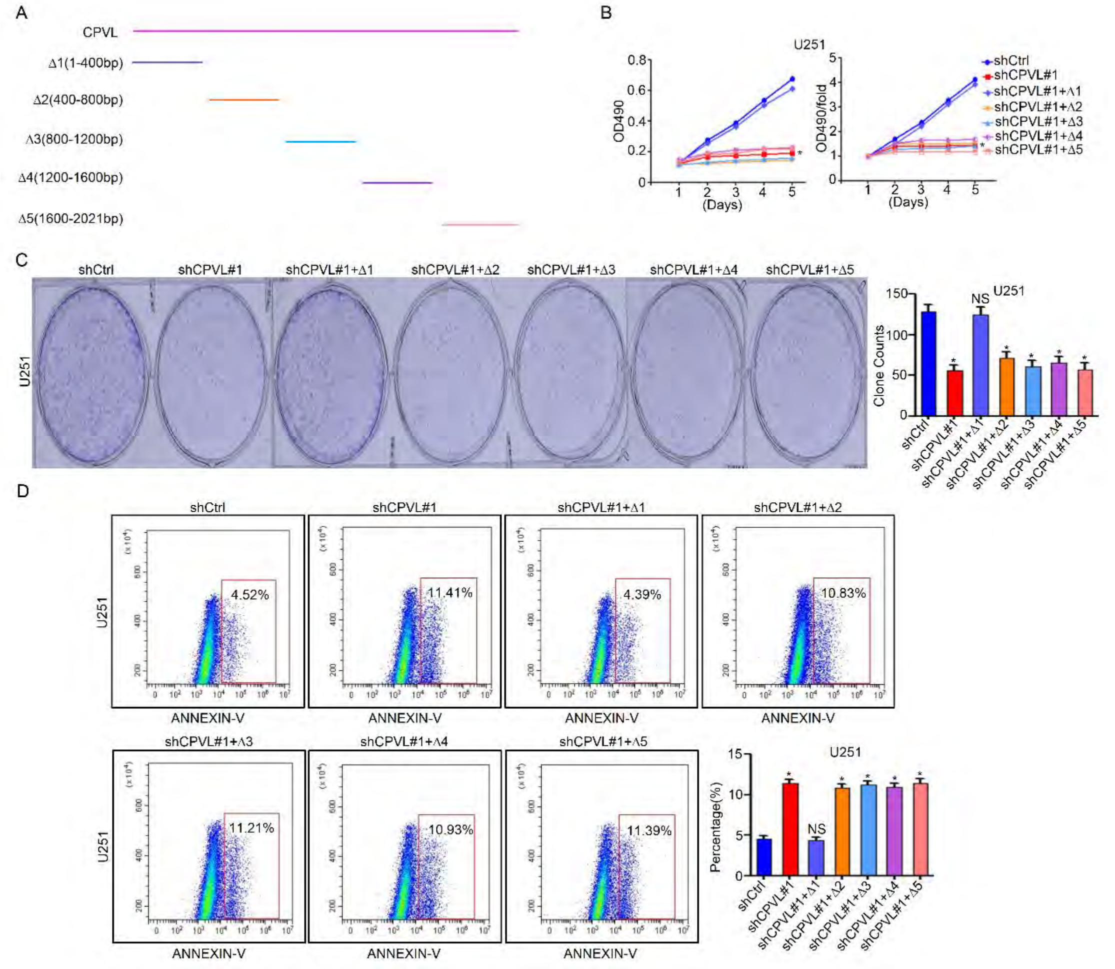

Supplemental Figure 5. Mutagenesis of the CPVL serine carboxypeptidase active site completely remove the oncogenic function of CPVL *in vitro*.

(**A**) A graphic illustration of the construction of mutagenesis of the different serine carboxypeptidase fragments (Δ1(1-400bp), Δ2(400-800bp), Δ3(800-1200bp), Δ4(1200-1600bp), Δ5(1600-2021bp)) of CPVL. (**B**) U251 cells were transfected with CPVL shRNA#1 or CPVL shRNA#1 plus mutagenesis of the different serine carboxypeptidase fragments (Δ1(1-400bp), Δ2(400-800bp), Δ3(800-1200bp), Δ4(1200-1600bp), Δ5(1600-2021bp)) of CPVL cDNA and the cell proliferation rates were investigated

by MTT assays. (**C**) U251 cells were transfected with CPVL shRNA#1 or CPVL shRNA#1 plus mutagenesis of the different serine carboxypeptidase fragments (Δ1(1-400bp), Δ2(400-800bp), Δ3(800-1200bp), Δ4(1200-1600bp), Δ5(1600-2021bp)) of CPVL cDNA and the cell proliferation capacities were investigated by colony formation assays. Representative pictures are shown on the left, and the number of colonies was counted on the right. (**D**) U251 cells were transfected with CPVL shRNA#1 or CPVL shRNA#1 plus mutagenesis of the different serine carboxypeptidase fragments (Δ1(1-400bp), Δ2(400-800bp), Δ3(800-1200bp), Δ4(1200-1600bp), Δ5(1600-2021bp)) of CPVL cDNA and the effect of cell apoptosis was detected by FACS assay. Representative profiles were shown on the left and the percentage of cells was statistically analyzed on the right. All experiments were repeated three times in triplicate. Bar graph data are presented as mean ± SD. One-way ANOVA with Dunnett's multiple comparisons test analyses were performed. *, $P<0.05$. NS, $P>0.05$.

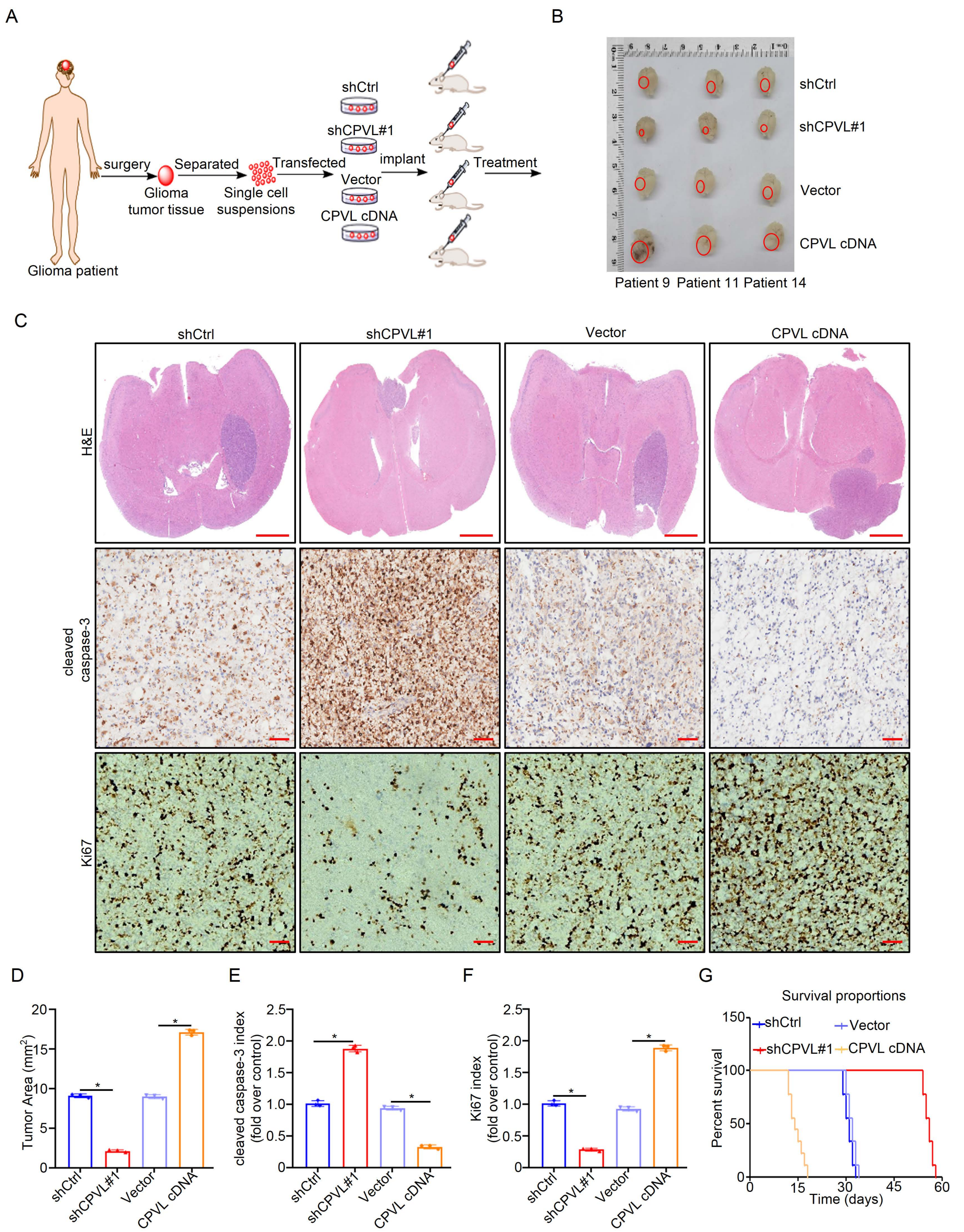

Supplemental Figure 6. CPVL silencing inhibits the tumorigenicity of glioma cells *in vivo*.

(**A**) A graphic illustration of the construction of intracranial glioma PDX mouse models. (**B**) Representative images of tumor growth. The tumor-bearing mice brain in the shCtrl, shCPVL#1, Vector, and CPVL cDNA

groups were harvested. The red ellipses show the main area where the carcinoma is found. The patients were clinically grade characterized (patients 9: Grade Ⅳ; patients 11: Grade Ⅳ; patients 14: Grade Ⅳ). (**C**) Representative H&E brain slices and IHC analysis of the indicated markers (cleaved caspase-3 and Ki67) in the intracranial glioma PDX tissues. (**D**) Tumor area was recorded in the tumor-bearing mice brain. (**E**) The expression levels of cleaved caspase-3 was statistically analyzed in the intracranial glioma PDX tissues. (**F**) The expression levels of Ki67 was statistically analyzed in the intracranial glioma PDX tissues. (**G**) Kaplan-Meier analysis of the mice intracranially implanted with shCtrl, shCPVL#1, Vector, and CPVL cDNA-transfected patient-derived primary glioma cells (n = 9 in each group).All data are presented as the mean ± SD. Two-tailed Student's test analyses were performed. *, P<0.05. Scale bars, HE: 400 μm (50× magnification); cleaved caspase-3 and Ki67: 100 μm (200× magnification) for panels.

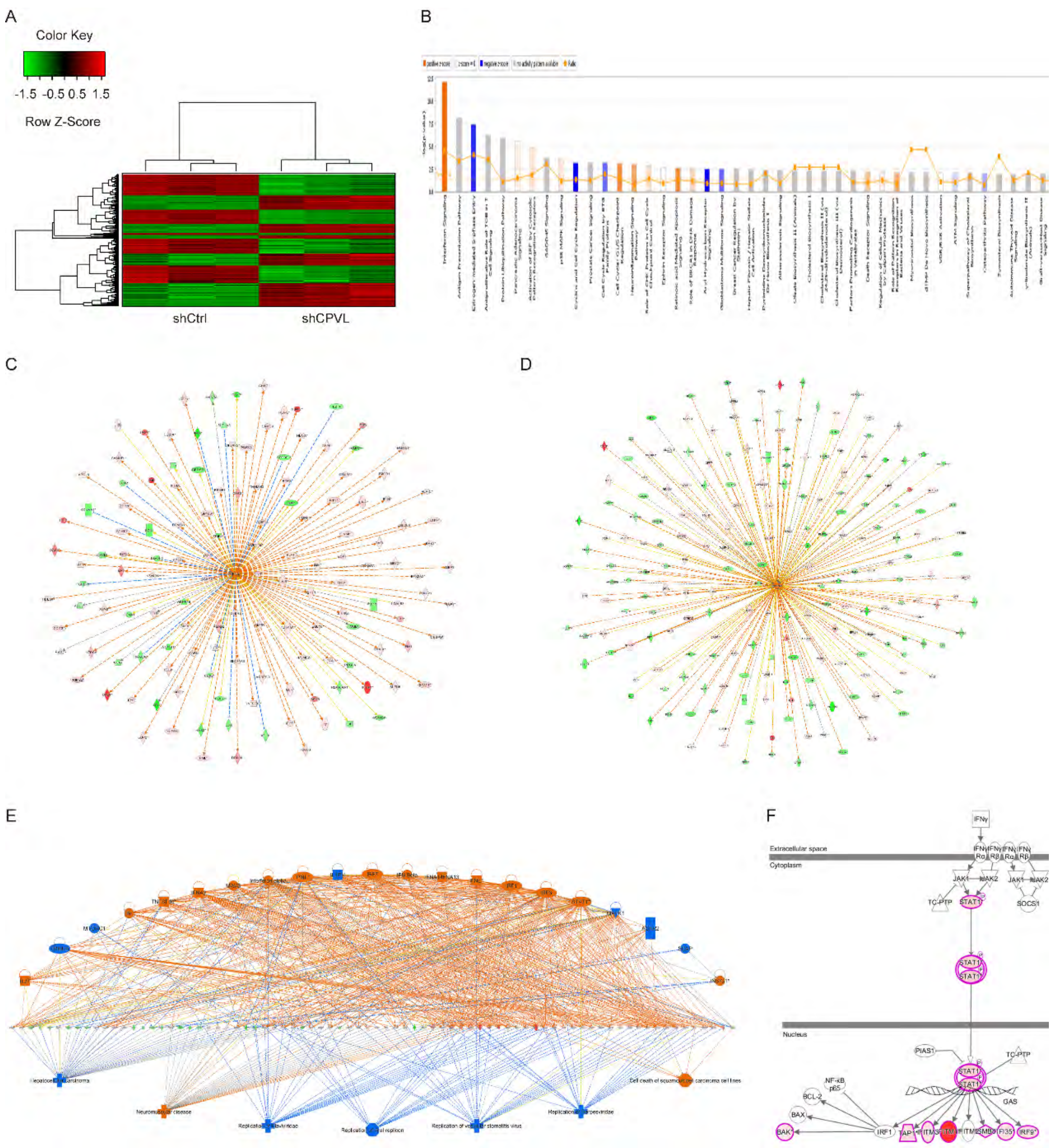

Supplemental Figure 7. Gene expression profile analysis reveals that the IFN-γ/STAT1 signaling pathway is a downstream target of CPVL that induces glioma cell apoptosis.

(A) Clustering analysis of differentially expressed genes in CPVL-silenced U251 compared to the control cells. Red indicates that the gene expression level is relatively up-regulated, green indicates that the gene expression level is relatively down-regulated, black indicates that there is no

significant change in the gene expression, and gray indicates that the signal intensity of the gene is not detected. (**B**) Gene-annotation enrichment analysis using IPA. Abscissa is the name of signal pathway, and ordinate is the significance level of enrichment. Orange indicates that the pathway is activated (z-score>0), blue indicates that the pathway is inhibited (z-score<0), and the depth of orange and blue (the absolute value of z-score) represents the degree of activation or inhibition. (**C**) IPA software predicts the activation or inhibition of the upstream regulator. The orange line indicates the consistent activation of upstream regulators and genes, the blue line indicates the consistent inhibition of upstream regulators and genes, the yellow line indicates the inconsistent expression trend between upstream regulators and genes, and the gray line indicates that there is no prediction information related to the expression status in the data set. (**D**) Disease and functional analysis based on IPA software. (**E**) The analysis of regulatory effects based on IPA software. The orange line indicates that the change of gene expression level has an activation effect on this function, the blue line indicates that the change of gene expression level has an inhibition effect on this function, the yellow line indicates that the change of gene expression level has an impact on this function which is inconsistent with the existing literature reports, and the gray line indicates that the regulatory relationship is unknown. (**F**) The expression change

trend of the IFN-γ signaling pathway various genes. Red represents that the gene is significantly up-regulated in the experimental results.

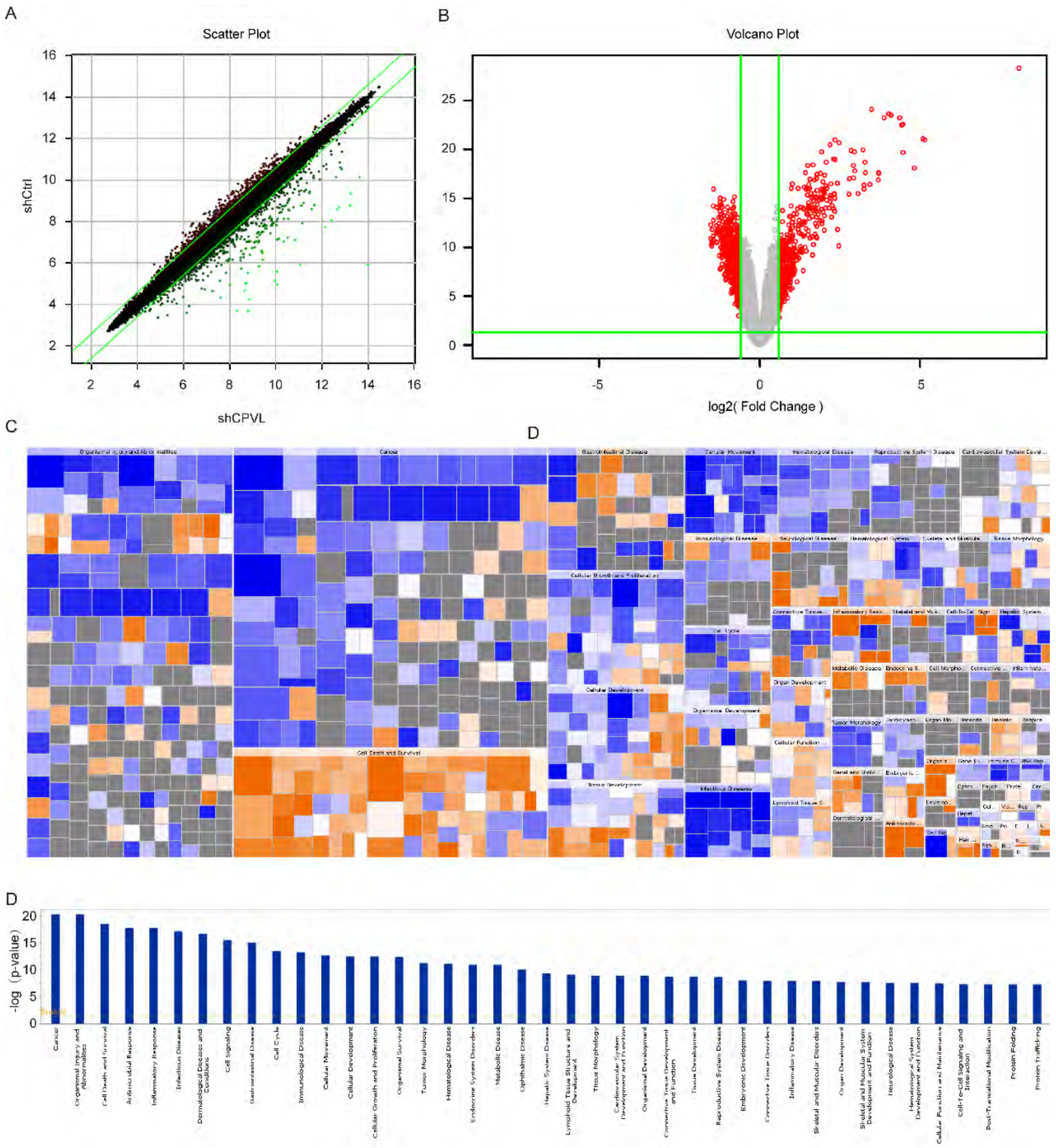

Supplemental Figure 8. The IFN-γ/STAT1 signaling pathway is a downstream target of CPVL that induces glioma cell apoptosis.

(**A**) Scatter plot of differentially expressed genes in CPVL-silenced U251 cells compared to the control cells. The parallel green solid line is the difference reference line, and the points within the reference line represent

the probe group with no significant change. Red points outside the reference line represent the probe group with relatively up-regulated expression in the control group, and green points represent the probe group with relatively up-regulated expression in the CPVL-silenced group. (**B**) Volcano plot of differentially expressed genes in CPVL-silenced U251 cells compared to the control cells. Red dots are significant differentially expressed genes and gray dots are non-significant differentially expressed genes. (**C**) Disease and function heat map of differentially expressed genes. Orange indicates that the disease or functional state is activated (z-score>0), blue indicates that the disease or functional state is inhibited (z-score<0), and gray indicates that the disease or functional state is not determined (z-score cannot be calculated). (**D**) Disease and function enrichment analysis statistics of differentially expressed genes. Abscissa is the name of signal pathway, and ordinate is the significance level of enrichment.

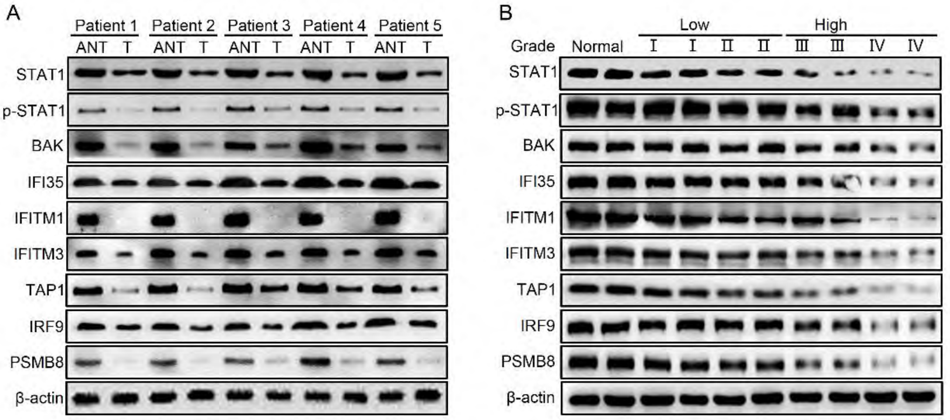

Supplemental Figure 9. Western blot analysis of the IFN-γ/STAT1 signaling pathway downstream response genes protein expression.

(**A**) Western blotting analysis of the IFN-γ/Stat1 signaling pathway downstream response genes protein expression in matched primary glioma tissues (T) and adjacent noncancerous tissues (ANT). The clinical grades of patients were characterized (patients 1: Grade Ⅱ; patients 2: Grade Ⅲ; patients 3: Grade Ⅰ; patients 4: Grade Ⅲ; patients 5: Grade Ⅳ). β-actin was used as a loading control. (**B**) Western blotting analysis of the expression of IFN-γ/STAT1 signaling pathway downstream response genes in glioma specimens at low and high clinical grades. β-actin was used as loading control.

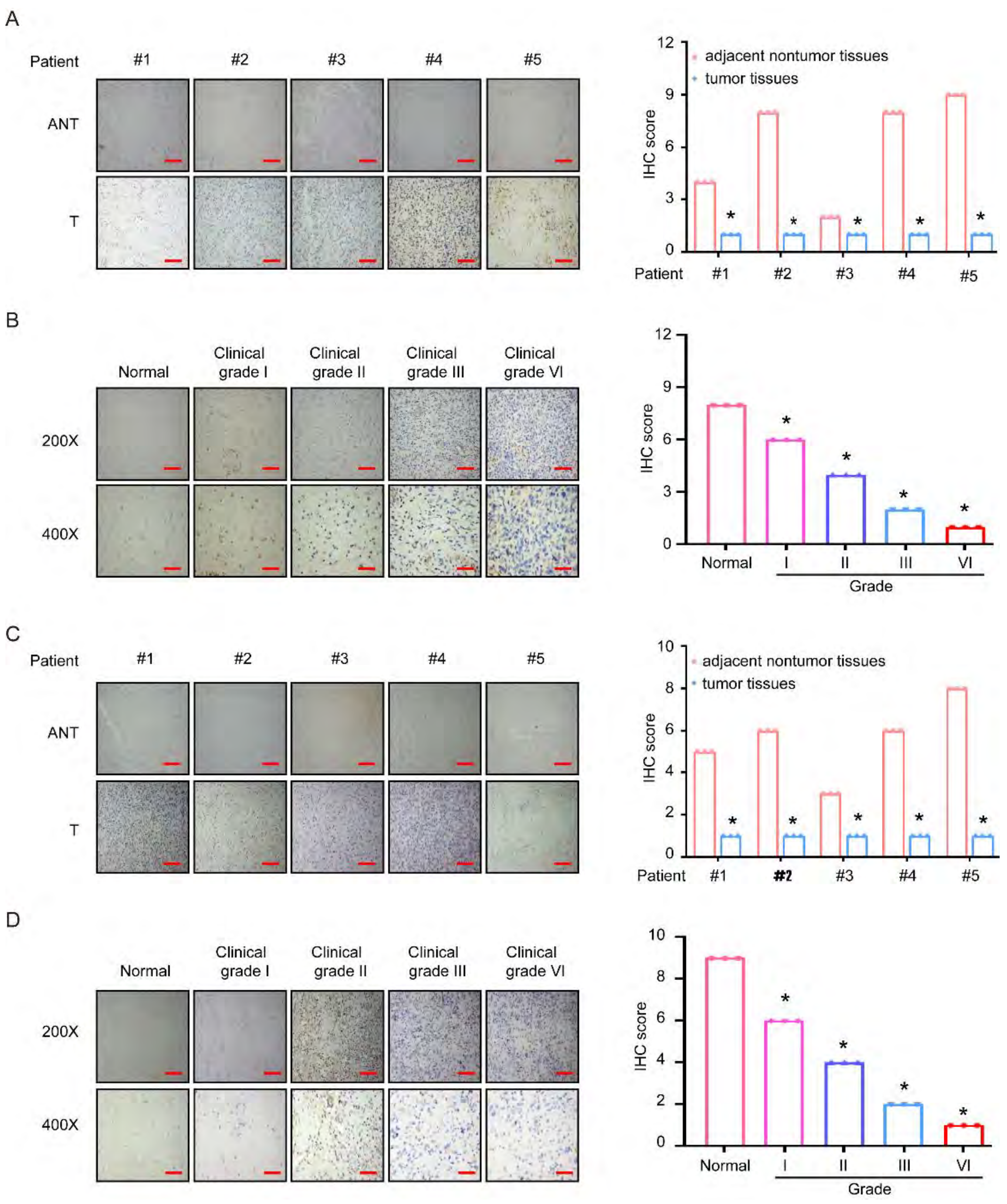

Supplemental Figure 10. IHC staining analysis of the expression of IFN-γ/STAT1 signaling pathway downstream response gene.

(**A**) IHC staining analysis of the expression of BAK in matched primary cancer tissues (T) and adjacent noncancerous tissues (ANT). Representative IHC images (left) and IHC score quantification (right) for BAK in tissue sections are shown. The clinical grades of patients were characterized (patients 1: Grade Ⅱ; patients 2: Grade Ⅲ; patients 3: Grade

Ⅰ; patients 4: Grade Ⅲ; patients 5: Grade Ⅳ). Scale bars, 100 μm (200× magnification) for panels. (**B**) IHC staining analysis of the expression of BAK in normal brain tissues and glioma tissues of different clinical grades. Representative IHC images (left) and IHC score quantification (right) for BAK in tissue sections are shown. Scale bars, 100 μm (200× magnification) for upper panels and 50 μm for lower panels (400× magnification). (**C**) IHC staining analysis of the expression of IRF9 in matched primary cancer tissues (T) and adjacent noncancerous tissues (ANT). Representative IHC images (left) and IHC score quantification (right) for IRF9 in tissue sections are shown. The clinical grades of patients were characterized (patients 1: Grade Ⅱ; patients 2: Grade Ⅲ; patients 3: Grade Ⅰ; patients 4: Grade Ⅲ; patients 5: Grade Ⅳ). Scale bars, 100 μm (200× magnification) for panels. (**D**) IHC staining analysis of the expression of IRF9 in normal brain tissues and glioma tissues of different clinical grades. Representative IHC images (left) and IHC score quantification (right) for IRF9 in tissue sections are shown. Scale bars, 100 μm (200× magnification) for upper panels and 50 μm for lower panels (400× magnification). One-way ANOVA with Dunnett's multiple comparisons test analyses were performed. *, $P<0.05$.

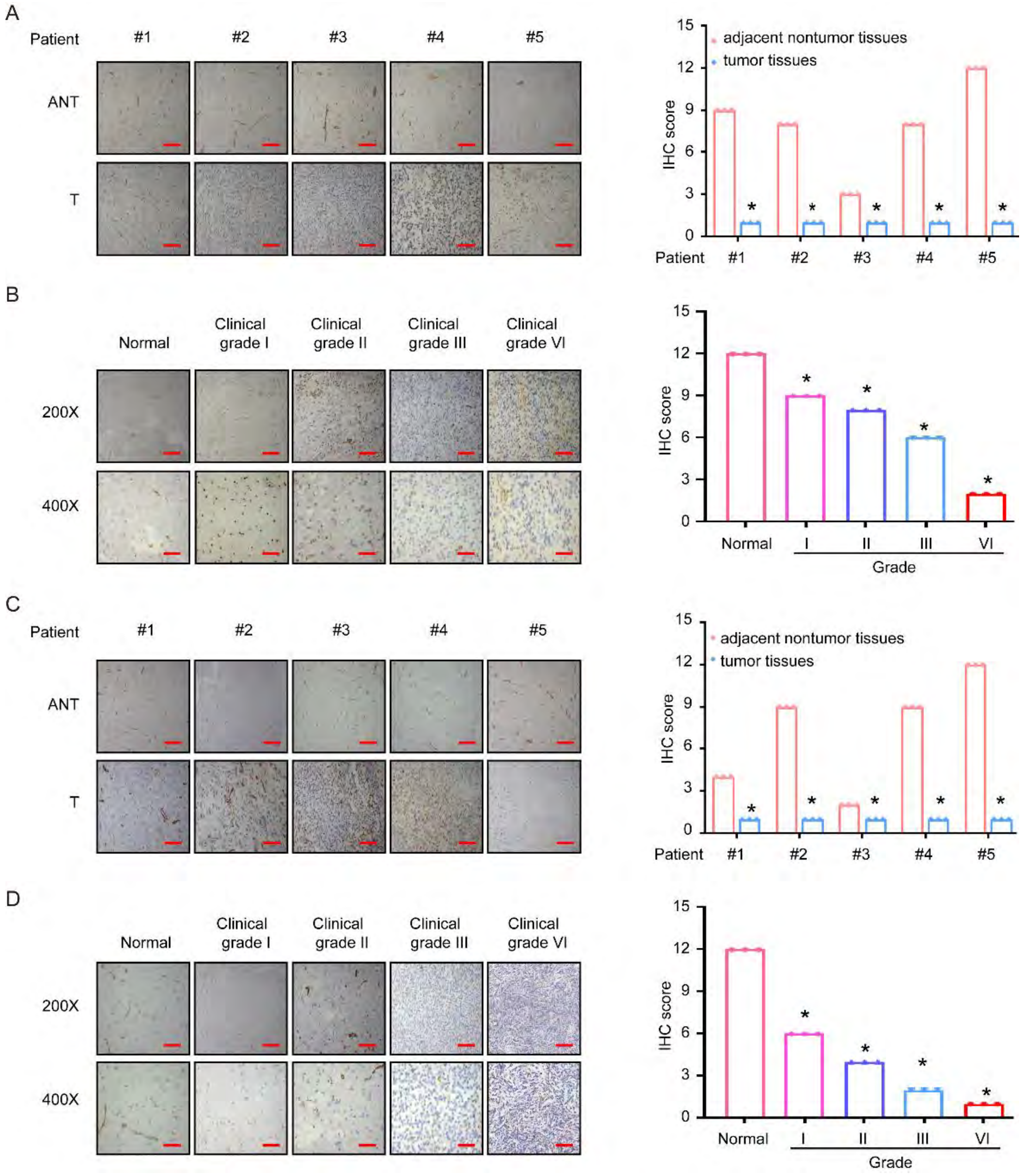

Supplemental Figure 11. IHC staining analysis of the expression of IFN-γ/Stat1 signaling pathway downstream response gene.

(**A**) IHC staining analysis of the expression of IFITM1 in matched primary cancer tissues (T) and adjacent noncancerous tissues (ANT). Representative IHC images (left) and IHC score quantification (right) for IFITM1 in tissue sections are shown. The clinical grades of patients were characterized (patients 1: GradeⅡ; patients 2: Grade Ⅲ; patients 3: Grade Ⅰ; patients 4: Grade Ⅲ; patients 5: Grade Ⅳ). Scale bars, 100 μm (200×

magnification) for panels. (**B**) IHC staining analysis of the expression of IFITM1 in normal brain tissues and glioma tissues of different clinical grades. Representative IHC images (left) and IHC score quantification (right) for IFITM1 in tissue sections are shown. Scale bars, 100 μm (200× magnification) for upper panels and 50 μm for lower panels (400× magnification). (**C**) IHC staining analysis of the expression of IFITM3 in matched primary cancer tissues (T) and adjacent noncancerous tissues (ANT). Representative IHC images (left) and IHC score quantification (right) for IFITM3 in tissue sections are shown. The clinical grades of patients were characterized (patients 1: Grade Ⅱ; patients 2: Grade Ⅲ; patients 3: Grade Ⅰ; patients 4: Grade Ⅲ; patients 5: Grade Ⅳ). Scale bars, 100 μm (200× magnification) for panels. (**D**) IHC staining analysis of the expression of IFITM3 in normal brain tissues and glioma tissues of different clinical grades. Representative IHC images (left) and IHC score quantification (right) for IFITM3 in tissue sections are shown. Scale bars, 100 μm (200× magnification) for upper panels and 50 μm for lower panels (400× magnification). One-way ANOVA with Dunnett's multiple comparisons test analyses were performed. *, P<0.05.

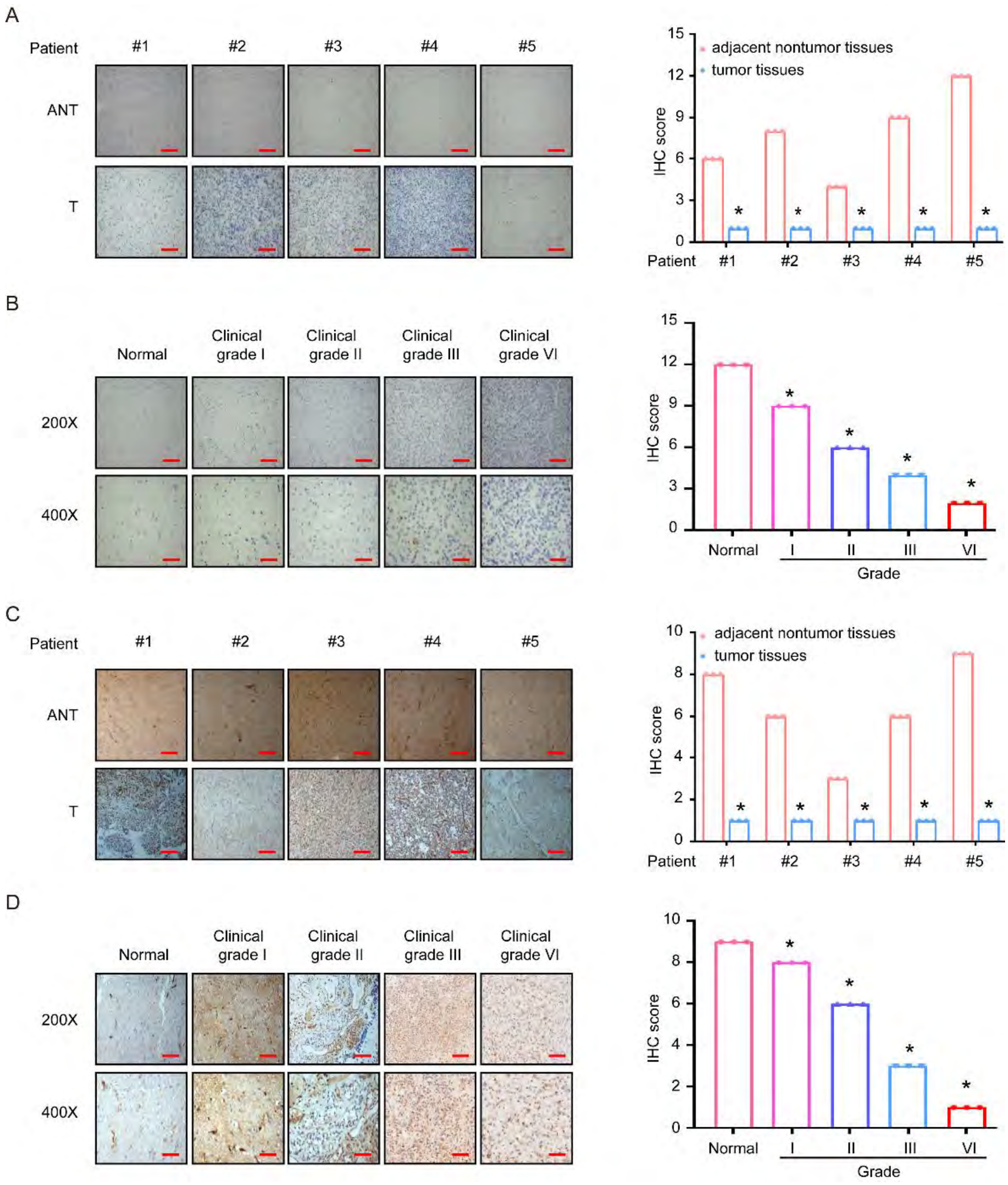

Supplemental Figure 12. IHC staining analysis of the expression of IFN-γ/Stat1 signaling pathway downstream response gene.

(**A**) IHC staining analysis of the expression of TAP1 in matched primary cancer tissues (T) and adjacent noncancerous tissues (ANT). Representative IHC images (left) and IHC score quantification (right) for TAP1 in tissue sections are shown. The clinical grades of patients were characterized (patients 1: Grade Ⅱ; patients 2: Grade Ⅲ; patients 3: Grade

Ⅰ; patients 4: Grade Ⅲ; patients 5: Grade Ⅳ). Scale bars, 100 μm (200× magnification) for panels. (**B**) IHC staining analysis of the expression of TAP1 in normal brain tissues and glioma tissues of different clinical grades. Representative IHC images (left) and IHC score quantification (right) for TAP1 in tissue sections are shown. Scale bars, 100 μm (200× magnification) for upper panels and 50 μm for lower panels (400× magnification). (**C**) IHC staining analysis of the expression of PSMB8 in matched primary cancer tissues (T) and adjacent noncancerous tissues (ANT). Representative IHC images (left) and IHC score quantification (right) for PSMB8 in tissue sections are shown. The clinical grades of patients were characterized (patients 1: Grade Ⅱ; patients 2: Grade Ⅲ; patients 3: Grade Ⅰ; patients 4: Grade Ⅲ; patients 5: Grade Ⅳ). Scale bars, 100 μm (200× magnification) for panels. (**D**) IHC staining analysis of the expression of PSMB8 in normal brain tissues and glioma tissues of different clinical grades. Representative IHC images (left) and IHC score quantification (right) for PSMB8 in tissue sections are shown. Scale bars, 100 μm (200× magnification) for upper panels and 50 μm for lower panels (400× magnification). One-way ANOVA with Dunnett's multiple comparisons test analyses were performed. *, $P<0.05$.

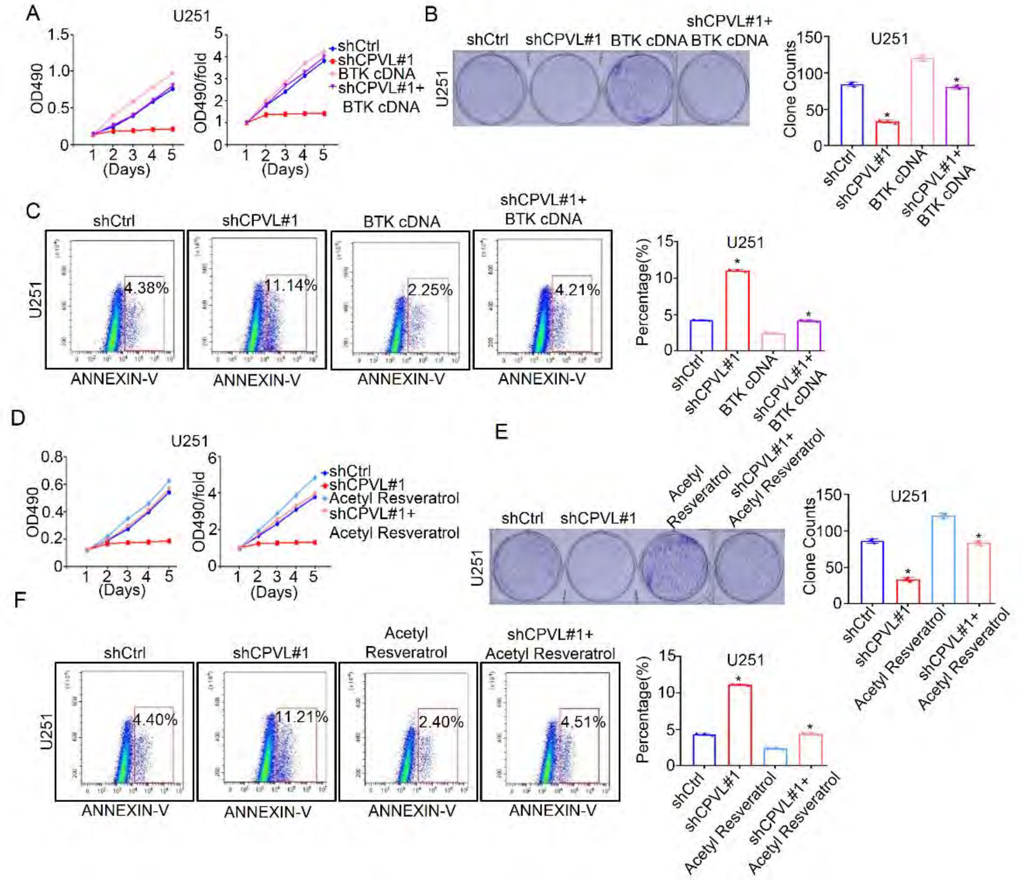

Supplemental Figure 13. Rescue experiments verify that CPVL regulates glioma progression *via* the STAT1 phosphorylation through p300-mediated STAT1 acetylation.

(**A**) U251 cells were transfected with CPVL shRNA or CPVL shRNA plus BTK cDNA compared to the control cells and the cell proliferation rates were investigated by MTT assays. (**B**) U251 cells were transfected with CPVL shRNA or CPVL shRNA plus BTK cDNA compared to the control cells and the cell proliferation capacities were investigated by colony formation assays. Representative pictures are shown on the left, and the number of colonies was counted on the right. (**C**) U251 cells were transfected with CPVL shRNA or CPVL shRNA plus BTK cDNA

compared to the control cells and the effect of cell apoptosis was detected by FACS assay. Representative profiles were shown on the left and the percentage of cells was statistically analyzed on the right. (**D**) MTT assays were used to investigate the proliferation rates in acetylation activator Acetyl Resveratrol treatment on CPVL-silenced U251 cells compared to the control cells. (**E**) Colony formation assay was used to investigate the proliferation capacity of acetylation activator Acetyl Resveratrol treatment on CPVL-silenced U251 cells compared to the control cells. Representative pictures are shown on the left, and the number of colonies was counted on the right. **(F)** FACS assay was used to detect the effect of acetylation activator Acetyl Resveratrol treatment on cell apoptosis in CPVL-silenced U251 cells compared to the control cells and the effect of cell apoptosis was detected by FACS assay. Representative profiles were shown on the left and the percentage of cells was statistically analyzed on the right. Experiments were conducted in triplicate. Bar graph data are presented as the mean ± SD. Two-tailed Student's test analyses were performed. *, $P<0.05$.

Supplemental table legends

Supplemental Table 1. The clinical information of 179 glioma patients. The table includes the 179 glioma patients' sex, age, histopathological type, operation time, survival state, follow-up time, overall survival, disease free survival, pathological grade, and organization code.

Supplemental Table 2. The Real-time PCR primers.

The table includes the primers of Quantitative real-time PCR analysis.

Supplemental Table 3. The results of statistical analysis of the IHC staining.

The table includes the 179 glioma patients' histopathological type, pathological classification, IHC staining intensity, IHC positive rate, IHC score.

Supplemental Table 4. Prognostic analysis of gene signature in the TCGA database.

The table includes the Overall Survival of high CPVL-expressing (n=166) and low CPVL-expressing (n=166) glioma patients from the TCGA database.

Supplemental Table 5. Prognostic analysis of gene signature in the TCGA database.

The table includes the Progression Free Survival of high CPVL-expressing (n=166) and low CPVL-expressing (n=166) glioma patients from the TCGA database.

Supplemental Table 6. Prognostic analysis of gene signature in the TCGA database.

The table includes the Disease Free Survival of high CPVL-expressing (n=158) and low CPVL-expressing (n=158) glioma patients from the TCGA database.

Supplemental Table 7. The activation of the upstream regulator.

The table includes the activation of the upstream regulator. In this study, IFN-γ/Stat1 was predicted to be strongly activated (Z-score=8.064, Stat1 Expression Fold Change=5.359), and 108 uniformly activated genes were identified.

Supplemental Table 8. The Mass spectrometry analysis of CPVL-containing protein complex.

The table includes the detailed results of the Mass spectrometry analysis of CPVL-containing protein complex.
