# Supplementary material for: CPVL promotes glioma progression via STAT1 pathway inhibition through interactions with the BTK/p300 axis
Source: JCI Insight. 2021 Dec 22;6(24):e146362. doi: 10.1172/jci.insight.146362 (PMC8783677; doi:10.1172/jci.insight.146362)
Supplement: Supplemental table 2 [file jciinsight-6-146362-s156.pdf]

Supplementary Table 2. Primers used for PCR amplifications

Gene	Primer (5'–3')	
CPVL	Forward	TCAACCTGAACGGAATTGCTA
	Reverse	GAAGGATCACTTGTTAAGTCGC
IRF9	Forward	CCGAAAACTCCGGAACTGGG
	Reverse	GGCCTTGAAGAAGGCAGCAT
NF-κB	Forward	CGGCTGAGTCCTGCTCCTTCC
	Reverse	TGGAGGCTGCCTGGATCACTTC
IFITM2	Forward	AGACCTCCGTGCCTGACCATG
	Reverse	CGTCGCCAACCATCTTCCTGTC
IRF1	Forward	CACTGTCGCCATGTGCTGTCAG
	Reverse	TGCCACTCCGACTGCTCCAAG
BAK	Forward	GGACGACATCAACCGACGCTATG
	Reverse	AACAGGCTGGTGGCAATCTTGG
TAP1	Forward	ACAGCCAGTTACAGGTGGAGCAG
	Reverse	CCGGATAGCGCCTCCTTCCAG
IFITM3	Forward	GCATTCGCCTACTCCGTGAAGTC
	Reverse	ACTTGGCGGTGGAGGCATAGG
IFITM1	Forward	GATCAACATCCACAGCGAGACCTC
	Reverse	GACTTCACGGAGTAGGCGAATGC
PSMB8	Forward	GAGACAGCTATTCTGGAGGCGTTG
	Reverse	GTGCAGCAGGTCACTGACATCTG
IFI35	Forward	CTCGGTACTGGTGCTCAACATTCC
	Reverse	ACTCAGAGGTGAAGACTGCTAGGC
BCl-2	Forward	ACTGAGTACCTGAACCGGCATC
	Reverse	GGAGAAATCAAACAGAGGTCGC
BAX	Forward	AGTGTCTCAGGCGAATTGGC
	Reverse	CACGGAAGAAGACCTCTCGG
BTK	Forward	CCAATGGCTGCCTCCTGAACTAC
	Reverse	TCGGTGAAGGAACTGCTTTGACTC
GAPDH	Forward	TGACTTCAACAGCGACACCCA
	Reverse	CACCCTGTTGCTGTAGCCAAA
